# Intentional Insulin Omission (Diabulimia) in Patients with Insulin-Dependent Diabetes: An Eating Disorder? A Systematic Review

**DOI:** 10.3390/jcm15093518

**Published:** 2026-05-04

**Authors:** Maria Benedetta Anesini, Mario Pinto, Michela Bellezza, Georgios D. Kotzalidis, Tommaso Callovini, Silvia Montanari, Camilla Scialpi, Gabriele Sani, Lorenzo Moccia, Delfina Janiri

**Affiliations:** 1 Section of Psychiatry, Department of Neurosciences, Università Cattolica del Sacro Cuore, 00168 Rome, Italy; mbenedetta@hotmail.it (M.B.A.); michelabellezza14@gmail.com (M.B.); giorgio.kotzalidis@gmail.com (G.D.K.); t.callovini@gmail.com (T.C.); silvia.montanari.rm@gmail.com (S.M.); camilla.scialpi@libero.it (C.S.); lorenzomoccia27@gmail.com (L.M.); or delfina.janiri@policlinicogemelli.it (D.J.); 2 Section of Psychiatry, Department of Neurosciences, Head-Neck and Chest, Fondazione Policlinico Universitario Agostino Gemelli IRCCS, 00168 Rome, Italy; mario.pinto@guest.policlinicogemelli.it

**Keywords:** intentional insulin omission, diabulimia, type 1 diabetes mellitus, eating disorders, insulin restriction, Glycaemic control, HbA1c, diabetes-related distress

## Abstract

**Background/Objectives:** Intentional insulin omission (IIO), commonly referred to as diabulimia, is a high-risk behavioural phenomenon observed mainly in adolescents and young adults with type 1 diabetes mellitus (T1D). Defined as the deliberate reduction or omission of insulin to influence body weight, IIO lies at the intersection of metabolic management and eating disorder psychopathology. Despite serious health risks, including diabetic ketoacidosis, microvascular complications, and increased mortality, it remains under-recognised due to stigma, diagnostic ambiguity, and overlap with routine diabetes self-management. This review aimed to examine the prevalence, psychological mechanisms, and clinical consequences of IIO. **Methods:** On 1 April 2026, we conducted a systematic search of PubMed, Scopus, APA PsycInfo/PsycArticles and Cinahl for studies investigating intentional insulin omission and related metabolic and psychological outcomes in T1D. Clinical and epidemiological studies assessing prevalence, risk factors, and interventions were included. **Results:** Twenty-nine studies met the inclusion criteria. Prevalence estimates ranged from 20% to 45%, with higher risk among females, adolescents, and individuals experiencing diabetes-related distress or body dissatisfaction. Psychological factors—including fear of weight gain, emotion dysregulation, depressive symptoms, and identity conflicts—were associated with IIO onset and maintenance. IIO was consistently linked to poor glycaemic control, elevated HbA1c levels, and adverse metabolic and psychological outcomes. Screening tools such as the Diabetes Eating Problem Survey–Revised (DEPS-R) may support early identification, while effective management requires integrated multidisciplinary care. **Conclusions:** Although IIO is not formally classified as an eating disorder in current diagnostic systems, it shares important psychopathological features with eating disorders and may represent a diabetes-specific disordered eating behaviour with life-threatening consequences.

## 1. Introduction

Disordered eating behaviours (DEBs) occur more frequently among adolescents and young adults with diabetes mellitus than in the general population, and they include binge eating and purging behaviours linked to insulin restriction or omission, a hazardous strategy for calorie elimination via intentional hyperglycaemia and glycosuria [1]. It is calculated that around 7% of patients dependent on insulin skip their doses [2]. This heightened vulnerability may be partly attributable to the intensive demands of diabetes self-management, which encompass detailed meal planning, strict portion control, and ongoing monitoring of food intake, particularly carbohydrate consumption, in relation to insulin dosing. Such requirements may increase susceptibility to food-related difficulties among individuals with diabetes [3]. Intentional Insulin Omission (IIO) as a weight-control strategy represents a central component in the understanding and management of DEBs, and is more commonly observed among individuals with type 1 diabetes mellitus (T1D). Therefore, inappropriate insulin use should be interpreted within the broader framework of eating disorders (EDs) rather than solely as a problem of diabetes management [4]. In this context, diabulimia has emerged as a newly defined diabetes-specific eating disorder (DSED), characterised by the intentional skipping of insulin doses or the administration of insufficient insulin with the purpose of losing weight [5]. This term has yet to receive validated diagnostic criteria, although the DSM-5/DSM-5-TR recognises insulin restriction as an analogue of purging behaviour.

Stuart Brink coined the term diabulimia to describe young people with Type 1 diabetes who learned that skipping insulin doses allowed them to binge without weight gain [6]. These young people are mostly female and expose themselves to chronic hyperglycaemia, glycosuria, elevated glycated haemoglobin levels, and increased muscle and fat catabolism with consequent failure of energy supply. Upon minimally stressful conditions, ketoacidosis and severe dehydration may ensue in rapid metabolic imbalance that may threaten life and require emergency intervention [7]. The term is pseudo-Greek, and its etymology, from διά (through) and βουλιμία (greed for food), makes little sense. It has been suggested that it is a blend of diabetes plus bulimia and indicates the coexistence of the two conditions [8], but again, diabulimia has nothing to do with bulimic behaviour; rather, it is related to a restrictive style of eating. Due to this, for diabulimia, we will mainly use here the more precise term IIO.

Diabulimia remains challenging to diagnose due to the absence of specific diagnostic criteria in medical and psychiatric guidelines, which forces healthcare professionals to rely largely on clinical judgment [9]. Although it is not officially recognised as a distinct diagnostic category in the DSM-5 or ICD-10, diabulimia represents a high-risk behavioural pattern that significantly increases the risk of both acute and long-term diabetes-related complications, including diabetic ketoacidosis, retinopathy, neuropathy, and premature mortality [4].

The often-hidden nature of the behaviour and the associated stigma contribute to limited clinical identification. In this context, diabulimia emerges as a complex and hybrid condition, situated at the boundary between metabolic and psychiatric pathology, requiring an integrated approach that involves multidisciplinary specialist expertise. However, the lack of formal diagnostic recognition in major classification systems represents a significant obstacle to the standardisation of research and the development of structured therapeutic pathways. Considering these critical issues, the present review aims to systematically synthesise the evidence currently available on diabulimia, with the goal of providing a conceptual framework useful for clinical practice.

## 2. Materials and Methods

On 1 April 2026, we searched four databases to identify literature focusing on IIO. For PubMed, we used diabulimia[ti] OR “insulin restriction”[ti] OR (skipping[ti] AND insulin[ti]) OR “insulin omission”[ti]; for Scopus, we used TITLE-ABS-KEY (diabulimia OR insulin restriction OR skipping insulin OR insulin omission); and for APA PsycINFO/PsycARTICLES and CINAHL, we used TI diabulimia OR TI insulin restriction OR TI skipping insulin OR TI insulin omission. We also searched the ClinicalTrials.gov sites for Condition/Disease: diabulimia OR Other terms: intentional insulin omission. All authors conducted the searches and compared their results. Resulting documents were retrieved and each characterised for eligibility through Delphi encounters in which all authors participated and discussed until reaching complete consensus. Study selection was performed through consensus meetings involving all authors. Given the full-team consensus approach adopted and the authors’ previous experience with high interrater agreement in similar review procedures (Cohen’s κ = 0.89), formal reassessment of interrater reliability was not performed in the present study. No more than two Delphi rounds were required to reach complete consensus; hence, no controversies had to be resolved by supervisors. In conducting this review, we adopted the *P*referred *R*eporting *I*tems for *S*ystematic reviews and *M*eta-*A*nalyses (PRISMA) Statement [10]. Eligible were studies investigating IIO and providing data; excluded studies were labelled as shown in Supplementary Table S1. The PRISMA 2020 checklist has been included in the Supplementary Materials (Table S2). Reviews, including meta-analyses and guidelines, were excluded; however, their reference lists were hand-searched to identify additional eligible studies. Similarly, editorials, letters to the editor with no data, theoretical articles and viewpoints were termed as Opinions and were excluded. Case reports and case series (termed Cases), studies lumping different diagnostic categories without providing separate data for IIO, studies reporting data on the same sample or on overlapping samples (termed Overlap; only the one with the largest sample was kept), animal studies, studies involving no patients, studies not including patients with diabetes, studies not studying IIO (labelled No diabulimia), studies whose focus was not on IIO or not suitably reporting results for IIO (labelled Unfocused), and studies not related to the subject matter (termed Unrelated) were also excluded. We also removed duplicates among databases and labelled as duplicates (intradatabase) all corrections that were reported in a database as separate documents. We registered our review on The Open Science Framework (OSF) platform with the following doi: 10.17605/OSF.IO/M2ADC.

### Quality Assessment

The methodological quality and risk of bias (RoB) of the included observational studies were assessed using the Risk of Bias In Non-randomised Studies of Interventions tool, version 2 (ROBINS-I V2) [11]. This instrument is specifically designed to evaluate non-randomised and observational studies in which the exposure is not randomly assigned and allows for a structured assessment of bias across the main stages of the study design, conduct, and reporting [11]. ROBINS-I V2 was first developed in 2016 by Sterne et al. [12]; it evaluates six domains of potential bias: bias due to confounding (D1), bias in the classification of the intervention or exposure (D2), bias in selection of participants into the study (D3), bias due to missing data (D4), bias in measurement of outcomes (D5), and bias in selection of the reported result (D6). For each domain, studies were judged as having low, moderate, serious, or critical risk of bias, or no information, following the ROBINS-I V2 guidance. In the confounding domain (D1), a judgment of low risk of bias could be assigned even in the presence of concerns about residual or uncontrolled confounding, provided that the key confounders were considered and addressed to an acceptable extent. An overall risk of bias judgment is then derived for each study based on the highest level of risk identified across domains. Any disagreements during the RoB assessment were resolved through discussion and consensus among all authors.

## 3. Results

**Figure 1:**
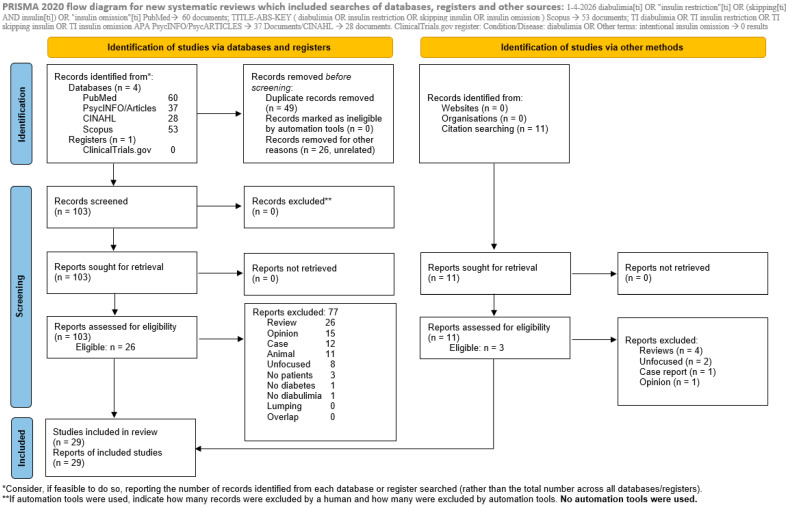
PRISMA flow-diagram of our review. From Page et al., 2021 [10]. This work is licensed under CC BY 4.0. To view a copy of this license, vist https://creativecommons.org/licenses/by/4.0/.

Eligible studies are shown in Table 1 and in Supplementary Table S1 in bold characters. On 1 April 2026, using the above-mentioned search strategies, PubMed identified 60 records, Scopus 53, APA PsycInfo/PsycArticles 37, and Cinahl 28. The ClinicalTrials.gov site yielded 0 results (Figure 1). Eleven more articles emerged from hand searching the reference lists of reviews and other interesting material, bringing the final count to a total of 189 studies (Supplementary Table S1). Included were 29 studies, while excluded were 160 records, i.e., 30 reviews, 16 opinion papers, 13 case reports or series, 11 animal studies, 3 for not including patients, 1 for not including patients with diabetes, 1 for not dealing with IIO/diabulimia, 10 for being unfocused and 26 for being unrelated to the subject of our search. There were 49 duplicates, while no study had population overlaps or lumped diagnoses in analysing data. The selection process and the reasons for exclusion are shown in Supplementary Table S1 and in Figure 1, where the PRISMA signal flow diagram is presented [10]. The first appearing study was published in March 1980 and was unrelated to our purposes; the last was published on 25 March 2026 and was eligible. Eligible studies spanned from October 1994 to 25 March 2026. Of the 29 eligible studies, 11 were conducted in the US; two each in the UK, Japan, Norway, and Türkiye; and one each in Nigeria, Israel, Germany, Belgium, Saudi Arabia, China–Taiwan, Greece, Brazil, France, and Canada. Overall, 8198 patients with T1D were involved; 25 of the designs were cross-sectional, and the other 4 were longitudinal (Table 1).

### Quality Assessment

The risk of bias assessment of the included observational studies using the ROBINS-I V2 tool is summarised in Table 2. Overall, most studies were judged to have a low-to-moderate risk of bias, with only a limited number of studies reaching a serious risk of bias in one or more domains. No study was classified as having a critical risk of bias.

For D1, most studies were judged to be at low risk of bias due to confounding, reflecting the identification and partial control of key confounders relevant to the relationship between insulin omission or restriction and psychosocial or clinical outcomes. A moderate risk of bias due to confounding was assigned to a small number of studies, primarily because of limited adjustment for potential psychosocial or metabolic confounders. A serious risk of bias was identified in one study, where substantial residual confounding could not be ruled out.

In D2, the majority of studies were judged to have a moderate risk of bias in the classification of the exposure, mainly due to reliance on self-reported measures of insulin omission or disordered eating behaviours without external validation. Several studies, particularly those using validated instruments or clearly defined exposure, were rated as having a low risk of bias in this domain.

For D3, while several investigations were judged to be at low risk, a moderate risk of bias was frequently assigned due to recruitment from specialised clinical settings or voluntary participation, which may limit representativeness. A serious risk of bias was identified in some studies, where selection mechanisms were likely related to both exposure and outcomes.

In D4, most studies demonstrated a low risk of bias due to missing data, as outcome data were largely complete or missingness was limited and unlikely to be related to both exposure and outcome. A moderate risk of bias was assigned in a few studies where incomplete reporting or attrition was present but not considered sufficient to substantially distort the findings.

For D5, the majority of studies were judged to have a low risk of bias in outcome measurement, particularly when validated psychometric instruments or objective clinical measures were used. A moderate risk of bias was assigned in studies where outcomes were exclusively self-reported and assessors were not blinded, increasing the potential for differential misclassification.

In D6, most studies were rated as having a low-to-moderate risk of bias in selective reporting. A moderate risk of bias was frequently assigned due to limited availability of pre-specified analysis plans or protocols. No evidence of selective reporting leading to a critical risk of bias was identified.

Based on the highest risk identified across domains, the overall risk of bias was judged as low for four out of 29 studies, moderate for the majority of the included studies (*n* = 20), and serious for five studies. Overall, the findings suggest that the available evidence is primarily affected by moderate methodological limitations, mainly related to exposure classification and participant selection, rather than by pervasive high-risk biases across multiple domains (see Figure S1 in Supplementary Material).

## 4. Discussion

The available literature on diabulimia is characterised by substantial variability, reflecting the lack of a clearly defined conceptual and diagnostic consensus. Despite this heterogeneity, the evidence consistently indicates that diabulimia is a high-risk and frequently concealed behavioural phenomenon, capable of gradually precipitating serious metabolic dysregulation and both acute and chronic diabetes-related complications. Yet, notwithstanding its potentially fatal outcomes, diabulimia continues to be under-recognised in clinical practice, largely due to its frequent clinical overlap with eating disorder features and routine diabetes management behaviours. As a result, diagnostic uncertainty often delays timely identification and appropriate intervention, allowing the condition to persist undetected over long periods.

### 4.1. An Underrecognised Public Health Issue

Since the earliest studies [13,14], the practice of IIO has been identified as a persistent, widespread, and substantially underestimated phenomenon. These early findings suggest that IIO is not a recently recognised behaviour, but rather a chronic, structural, and epidemiologically concerning problem in the management of T1D. Table 1 highlights that IIO and DSED are prevalent across diverse geographical contexts. This broad geographical distribution indicates that the behaviour is not confined to specific regions or cultural practices but rather represents a common and cross-cutting clinical issue, although low- and middle-income countries remain underrepresented in the literature. The available evidence consistently suggests that IIO is a highly prevalent behaviour, with reported rates ranging from 20% to 45%, depending on the population studied, age group, and assessment instruments employed. These figures may be unreliable, as not all studies assess the motivations for skipping doses, and some of them include unintentional or involuntary (i.e., of which patients are unaware) skipping among cases, thus inflating the prevalence rate. In line with these findings, Peyrot et al. [20] reported that 57% of participants had omitted an insulin injection at least once despite being aware of its necessity, while 20% acknowledged omitting injections on a regular basis. Comparable findings were later reported in adolescent samples. More recent studies using standardised screening tools such as the Diabetes Eating Problem Survey–Revised (DEPS-R) [39] suggest that IIO and the risk of diabulimia affect a substantial proportion of adolescents and young adults with T1D. Reported prevalence rates range between 23.9% and 31.8%, suggesting that approximately one-quarter to one-third of this population exhibits IIO [5,31,33]. Higher prevalence rates have been observed among younger individuals, those with lower income, and unexpectedly, those with higher levels of education. Conversely, a lower likelihood has been associated with older age, higher income, and the adoption of dietary habits considered healthy [20]. In addition, individuals with disabilities have been shown to have a lower frequency of IIO, likely due to increased caregiving support and supervision, which may facilitate greater adherence to treatment regimens [20]. The risk of omitting injectable therapy is increased in the presence of specific factors related to the complexity of the therapeutic regimen and its impact on daily life, independent of diabetes type and other clinical or sociodemographic variables. A high frequency of daily injections; interference of treatment with work, social, and family activities; the need to plan daily routines around insulin administration; perceived injection-related pain; and discomfort or embarrassment in social contexts have all been identified as key determinants of treatment adherence [20]. By contrast, factors such as fear of hypoglycaemia, the type of delivery device used (pen versus syringe), ease of use, or the time required to administer injections do not appear to play a major role in determining the prevalence of IIO [20]. Rather, it is the overall burden of treatment on patients’ daily lives that emerges as a far more influential factor than the technical characteristics of the device or other isolated treatment-related variables. Although variability in reported prevalence rates is likely attributable to methodological differences across studies, the available evidence converges in showing that IIO is considerably more widespread than is typically recognised in routine clinical practice. Estimating the true prevalence is further complicated by the fact that many patients do not disclose them to clinicians due to shame and stigma, leading to substantial clinical underestimation and a significant gap in diagnostic recognition [29,34].

### 4.2. Developmental Stages and Windows of Vulnerability

Adolescence represents a particularly critical developmental period for the onset of DEB [22,23], although these behaviours are not confined to this phase and may be observed across all age groups [13]. IIO may emerge in adulthood or reoccur after periods of remission [21,29]. The prevalence of DEBs increases among girls between 17 and 19 years of age and is further elevated in the presence of weight gain. During this developmental period, physical appearance comparisons through social media have been shown to be significantly associated with DSED and dietary restraint [36]. Such comparisons contribute to lower weight satisfaction and represent one of the strongest correlates of dietary restraint, as well as a relevant factor in the adoption of DSED. Additional risk factors include family conflict, negative body image perception, psychological distress, and increasing autonomy, particularly during developmental transition phases [5,27]. Notably, more than half of girls with obesity exhibit DEBs and omit insulin administration after meals [22]. Among the main predictors of IIO are diabetes-specific distress and fear of weight gain associated with insulin therapy [13]. Diabetes constitutes a unique clinical context in which body weight control, adherence to dietary prescriptions, and maintenance of adequate glycaemic levels are closely intertwined, rendering individuals T1D particularly vulnerable to dysfunctional eating behaviour patterns. Partially contrasting with these findings, Ackard et al. [18] reported that young adults with T1D exhibit, overall, a more adaptive eating behaviour profile than their peers without diabetes, characterised by lower body weight dissatisfaction, reduced engagement in disordered eating practices (vomiting, use of diuretics or laxatives, and IIO), and greater regularity in the consumption of main meals. Weight changes occurring around the time of diagnosis and during the initial phase of insulin therapy may contribute to body dissatisfaction [37]. This pattern may be attributed to the need for a structured and closely monitored daily dietary regimen imposed by diabetes management, which may foster greater awareness in meal planning and exert a protective effect on the relationship with food and body image.

Since the earliest investigations, the literature has consistently documented a female predominance in DEB [13,14]. In one of the earliest observational studies, Polonsky et al. [13] documented regular IIO in 30.5% of women. Khan and Montgomery [14] found that 22.9% of young women reported IIO at least once per month. Longitudinal studies in adult populations have reported comparable prevalence rates, with more than 30% of women engaging in IIO [16,21,28]. Prevalence rates are even higher when samples include overweight adolescents [32]. In paediatric samples, IIO appears to be particularly prevalent among females. In a national epidemiological study, Wisting et al. [22] observed that 36.8% of adolescent girls reported insulin restriction and 26.2% reported complete omission of doses. Although many studies confirm a higher prevalence of these behaviours in women, IIO appears to occur at relatively similar rates in males and females [25,28,33]. In this regard, Ackard et al. [18] found that 10.3% of girls and 1.4% of boys reported deliberately skipping insulin doses, while 7.4% of girls and 1.4% of boys reported reducing insulin as a strategy for weight control. Moreover, girls with diabetes showed lower rates of dieting, fasting, and dietary restriction compared with their non-diabetic peers, suggesting a potential protective effect of therapeutic management on eating control. Conversely, boys with diabetes reported lower levels of physical activity and reduced consumption of fruits and vegetables compared with their non-diabetic counterparts, indicating the presence of gender-specific behavioural differences. Overall, these clinically hazardous behaviours point to the existence of diabetes-specific weight control strategies and underscore a particular vulnerability in adolescents of both sexes, even in the context of generally more regular eating patterns.

More recent studies, however, have reported attenuated or absent gender differences, particularly during adolescence [31,33,34]. Despite this, women continue to exhibit greater clinical and psychopathological severity, especially with respect to IIO as a means of weight control [22,36]. Consistent with this greater severity, women who discontinue IIO show significant improvements in self-care behaviours, diabetes-related distress, and disease management difficulties, as well as reductions in eating disorder-related psychological symptoms. At the same time, body mass index (BMI) remains stable, indicating that cessation of IIO does not result in weight gain. This finding is clinically relevant, as it challenges the fear of weight gain associated with insulin treatment adherence and reinforces the importance of targeted interventions aimed at improving both metabolic and psychological outcomes [21].

### 4.3. Metabolic Consequences and Clinical Implications

The scientific literature highlights a bidirectional relationship between DEB and metabolic control as measured by glycated haemoglobin (HbA1c) levels [28]. Specifically, the presence of DEB is associated with poorer glycaemic control, resulting in increased HbA1c values. Conversely, elevated HbA1c levels appear to promote DEB, likely mediated by psychological and behavioural mechanisms related to distress, frustration, and the burden of daily disease management. This reciprocal interaction may contribute to the development of a vicious cycle in which poor glycaemic control and DEB mutually reinforce each other, with potentially detrimental effects on long-term clinical outcomes. Evidence indicates that IIO is consistently associated with higher HbA1c values and poor glycaemic control [15,16,22,25,33]. Moreover, the more frequently insulin is omitted or reduced, the higher the mean blood glucose levels and the greater the time spent in hyperglycaemia, leading to further deterioration of metabolic status [24]. IIO has been shown to be a stronger predictor of poor metabolic control than other compensatory behaviours, including binge eating and purging [15]. Also, IIO is associated with severe medical consequences, including a significant increase in hospitalisations for diabetic ketoacidosis (DKA) and an elevated risk of chronic complications, particularly diabetic retinopathy, neuropathy, and nephropathy [13,16,22,25]. Longitudinal studies have further demonstrated an association between IIO and an increased incidence of microvascular complications, foot-related problems, and mortality [16,21]. Consistent with these findings, the presence of DEBs is correlated with higher HbA1c and BMI values, as well as a greater frequency of DKA-related hospitalisations and severe hypoglycaemic episodes [33]. Increased rates of DKA and hospitalisations have been reported particularly among adolescents with IIO and DEB [25,29,34]. Adolescence involves major physical, hormonal, and psycho-emotional changes that hinder optimal disease management [4]. The duration of IIO represents a more robust predictor of complication development than baseline HbA1c levels [17], underscoring the cumulative and progressive effect on organ damage. Higher levels of anxiety and depressive symptoms have also been frequently reported in patients with ED who engage in IIO [15].

A biological mechanism mediated by the gut microbiota has also been hypothesised. Igudesman’s et al. study [32] showed that DEB and IIO are associated with unfavourable alterations in gut microbiota composition, including a reduction in *Anaerostipes* associated with increased DEB and a decrease in *Lachnospira* associated with IIO. Both bacterial genera are producers of short-chain fatty acids (SCFAs), key molecules involved in glycaemic regulation, inflammation modulation, and the maintenance of overall metabolic health, suggesting a potential microbiota-mediated contribution to the metabolic deterioration observed in these patients.

IIO is also associated with up to a threefold increase in mortality risk, with an average reduction in life expectancy of approximately 13 years, establishing it as a reliable predictor of mortality [16]. In the same study, deceased women exhibited significantly higher HbA1c levels, higher BMI, and more severe ED symptomatology, which was associated with more frequent IIO. Importantly, intentional non-adherence is not limited to insulin administration but also extends to oral hypoglycaemic agents: 70.2% of patients voluntarily omit prescribed doses, suggesting a behavioural issue that cuts across different antidiabetic treatment modalities [19]. Overall, these dysfunctional behaviours can be conceptualised as part of the spectrum encompassed by the entity known as diabulimia.

### 4.4. Psychological and Psychopathological Mechanisms Involved

Beyond the physical consequences of poor glycaemic control, diabulimia is also linked to a considerable burden of psychological morbidity, particularly diabetes-related distress [40]. The relentless demands of diabetes self-management impose a significant psychological burden, frequently resulting in anxiety, depressive symptomatology, and emotional exhaustion [41]. Patients perceived diabetes as a central component of their identity. This identity integration appears to be associated with increased psychological burden, as patients commonly report fear of both acute and long-term diabetes-related complications and feelings of being overwhelmed by the continuous demands of treatment [42]. This sustained psychological strain, combined with a heightened need for control over body weight and conformity to societal or personal physical appearance standards, may contribute to the onset and maintenance of DSED, including the deliberate omission or restriction of insulin for the purpose of weight loss. These practices cannot be interpreted solely as problems of treatment adherence, but rather as complex behaviours embedded in broader frameworks of emotional fragility, body-related distress, and difficulties in affect regulation. About one-third of patients with IIO have a co-occurring ED [38]. Khan and Montgomery [14] showed that key aspects of diabetes treatment—such as constant glycaemic monitoring, dietary restrictions, and insulin-related weight changes—can foster body dissatisfaction and restrictive eating patterns. In line with this, girls with T1D report a greater drive for thinness, body dissatisfaction, and more frequent purging behaviours than healthy peers. IIO thus reflects a psychological profile marked by low self-esteem, poor perceived treatment efficacy, reduced bodily awareness, and heightened sensitivity to food-related cues [14]. Within this framework, fear that improved glycaemic control will lead to weight gain emerges as a core issue and represents the strongest predictor of whether insulin restriction is discontinued or maintained over time [21,22]. Body weight control is the primary driver of IIO, although not the only one [14,29]. Polonsky et al. [13] reported that approximately 50% of participants used IIO as a weight-control strategy, also showing a higher prevalence of ED and elevated levels of psychological and diabetes-specific distress. In the exclusively female sample, achieving good glycaemic control was associated with a marked fear of weight gain, which in turn led to poorer adherence to treatment using IIO for weight loss purposes. Those who misuse insulin to control weight report significantly higher levels of body dissatisfaction than peers who do not engage in this behaviour, despite no differences in BMI [18]. This suggests that the motivation underlying insulin misuse is not related to actual overweight, but rather to psychological factors such as body perception and the emotional distress associated with body image. In this context, attitudes toward weight play a central role in maintaining IIO. The fear of weight gain associated with improved glycaemic control, combined with self-management difficulties and emotional distress, fuels a dysfunctional pattern in which weight becomes a measure of personal worth.

IIO thus appears to be a perceived but ineffective control strategy. A clinically relevant paradox emerges; women who continue or initiate IIO tend to gain weight over time, despite the explicit goal of preventing weight increase. This finding reinforces the hypothesis that restriction not only fails as a weight-control strategy but also contributes to the maintenance of a vicious cycle of insulin fear, thus impairing the management of diabetes. The resulting increased psychological distress has direct implications for clinical intervention and therapeutic education [21]. Moreover, DEB predict increasing levels of depression over time, thus being a psychological rather than exclusively medical risk factor [28]. The continuous and demanding nature of T1D management imposes a substantial emotional burden and is associated with an increased risk of mental health disorders [37]. Depression, anxiety, diabetes-related distress, and emotion dysregulation are strongly associated with IIO [27,30,35,36]. The presence of a psychiatric disorder is associated with an almost six-fold increase in the risk of DSED, with anxious–depressive symptomatology emerging as the main predictive factor [5]. Adolescents who intentionally omit insulin to control weight show higher levels of depression, anxiety, diabetes-related distress, and family conflict [27]. Compared with peers who do not omit insulin, they are also more likely to report significant depressive symptoms. This issue is frequently underreported by parents, who do not perceive the same level of distress reported by their children, increasing the risk of underestimating clinical severity. Risk is further elevated in the presence of divorced parents [5].

From an emotion dysregulation perspective, IIO may take on an impulsive form and be closely associated with acute emotional states, particularly before meals, such as anxiety, nervousness, guilt, self-disgust, sadness, anger, and frustration, as well as distress related to diabetes management [24]. Even mild but transient increases in negative effect are sufficient to double the likelihood of IIO. Furthermore, Merwin et al. [24] indicate that IIO is more strongly predicted by the subjective appraisal of the eating episode, such as the perception of having broken a rule (“no sweets”), having lost control, or having experienced guilt, rather than by the objective characteristics of the food consumed. Finally, the study by Beam et al. [30] showed that general insulin restriction (IR) and insulin restriction for weight control (IRWC) represent two distinct and uncorrelated behaviours with different clinical consequences. IR appears to be associated with poorer overall diabetes management, whereas IRWC is specifically related to higher HbA1c levels, indicative of impaired glycaemic control. Depressive symptoms show a strong association with both forms of restriction but play a central role in IRWC: once depression is controlled for, the relationship between insulin restriction for weight and emotion dysregulation loses significance, suggesting that depression may explain why adolescents limit insulin to influence body weight. In contrast, in general insulin restriction, emotion dysregulation plays a relevant role primarily in the presence of high levels of depressive symptoms.

### 4.5. Diabulimia as an Emerging New Clinical Entity

The intersection between insulin therapy and eating disorder pathology may give rise to a specific condition commonly referred to as diabulimia. This condition has been described as the world’s most dangerous eating disorder and occurs when people with T1D deliberately misuse insulin to control their weight, leading to potentially devastating complications [43]. The prevalence of diabulimia has been estimated at approximately 2% among preadolescent girls, increasing to 11–15% in adolescents and reaching 30–39% in young women aged 12–18 years, highlighting a marked age-related increase in risk [34]. Across various studies, its prevalence ranged from about 20% to almost 50%, with about twice the number of females being affected compared to males (45% vs. 26%) [44]. Although the term diabulimia is widely used in both clinical and research contexts, it lacks formal diagnostic recognition and may not adequately capture the full spectrum of insulin dysregulation behaviours observed in ED associated with T1D [4]. It is classified in the group of “drug abuse to ensure undefined eating disorder/weight loss” in the fifth edition of the Diagnostic and Statistical Manual of Mental Disorders (DSM-5) [31]. Therefore, IIO should be conceptualised not merely as a weight-control strategy, but within the broader framework of ED psychopathology. Despite the significant health risks, diabulimia remains largely underdiagnosed and underrecognised [45]. It predominantly affects young individuals, with a higher prevalence among females, and lies at the intersection of diabetes management, emotional regulation, and body image concerns, constituting a condition in which metabolic care and mental health are deeply interconnected [43]. However, the purported higher prevalence of IIO in female patients with T1D is not confirmed by several studies [5,31,33,38], but the fact that some studies were limited to female patients renders the observed gender differences unreliable. Major risk factors for the development of ED and DEB include adolescence, female sex, body dissatisfaction, and a dysfunctional family environment [4]. Adolescence represents a particularly critical period, during which bodily changes, the desire for autonomy in disease management, and difficulties in transitioning to adulthood may increase vulnerability to the development of diabulimia [9]. Moreover, the presence of a pre-existing ED prior to diabetes onset or a family history of EDs in first-degree relatives significantly increases the risk of IIO [9]. Prevalence estimates of IIO vary considerably depending on the assessment methodology employed. However, studies using DEPS-R—a measure considered one of the most psychometrically robust tools currently available for assessing insulin manipulation—have reported prevalence rates as high as 60.2% in samples of individuals with TD1M aged 13 to 55 years [46]. For clinical screening, a DEPS-R total score ≥ 20 has been proposed as a clinically relevant threshold to identify individuals at risk of DEB, poor glycaemic control, diabulimia, and body image disturbances [4]. Among clinical correlates most strongly associated with diabulimia are elevated HbA1c levels, increased emergency department visits and hospitalisations for diabetic complications, and the presence of MDD. Notably, individuals with depression exhibit an almost fivefold increased risk of developing behaviours consistent with diabulimia compared to those without depressive symptoms [34]. Depressive symptoms may undermine motivation and self-efficacy, leading to inconsistent glucose monitoring, insulin omission, and emotional eating. The association between depression and DEB is likely bidirectional: depression may increase vulnerability to DEBs, while feelings of guilt and frustration may, in turn, exacerbate depressive symptoms [37]. From a psychopathological standpoint, diabulimia represents a form of severe ED psychopathology. Symptom severity measured by the Eating Disorder Examination Questionnaire (EDE-Q) is comparable to that observed in clinically diagnosed eating disorders and significantly higher than normative population values [26]. IIO typically begins around 19 years of age, although a wide age range (12–45 years) has been reported, suggesting persistent and transdiagnostic vulnerability over time [29]. In some cases, IIO co-occurs with the use of weight-loss medications without medical indication [35]. The motivations underlying IIO are multifactorial and include weight control driven by fear of weight gain, excessive control and cognitive distortions of body image, rejection of the diabetic condition with difficulties in accepting a chronic illness and a desire for identity normalisation, as well as a self-harming function, whereby intentional hyperglycaemia may serve as emotional anaesthesia or self-punishment. These factors are compounded by precipitating and maintaining conditions such as trauma, abuse, bullying, family disruption, psychiatric comorbidities, and lack of psychosocial support, often exacerbated by shame, stigma, and secrecy surrounding the behaviour. A particularly critical issue concerns the relationship with healthcare providers. In fact, 65% of patients report never having disclosed IIO to their physician and avoiding contact with specialised support services [34]. Most individuals describe experiences of misunderstanding, judgment, and perceived lack of provider competence, with a reductionist approach focused exclusively on insulin adherence, failing to address the underlying psychological distress [29]. Finally, body dissatisfaction and concerns about weight and physical appearance represent additional significant risk factors [9], reinforcing the need for an integrated approach that incorporates metabolic, psychological, and relational dimensions in both the prevention and treatment of this complex condition.

### 4.6. Clinical Implications for Preventive Care

Even the presence of a single DEB has greater clinical relevance in young adults with T1D than in the general population, as it directly compromises glycaemic control and increases the risk of acute and chronic complications [18]. In the absence of intervention, DEBs are associated with significant deterioration in both physical and mental health [28]. In this context, IIO is often not detected by traditional ED questionnaires, yet it is crucial to identify it for accurate risk assessment and the timely initiation of appropriate therapeutic interventions [25]. Therefore, both ED and IIO should be assessed during the initial clinical evaluation. Regular screening is essential to enable early identification of diabulimia risk and timely diagnosis supported by psychiatric evaluation [31]. The introduction of simple screening questions into routine clinical practice, such as “I take less insulin than I should,” may facilitate the early identification of at-risk individuals and their referral to specialist support [16]. Routine screening for ED should be particularly emphasised among individuals with elevated BMI and persistently high HbA1c levels [5,33]. In this perspective, disease-specific tools such as the DEPS-R have proven effective in the early detection of DEB risk, showing a significant correlation between higher scores and poorer glycaemic control. This finding further supports the role of IIO as an important risk factor for the development of diabetes-related complications [5,22,28,33]. Several observational studies therefore recommend routine screening for DEBs during diabetes clinic visits [27,31], embedding this practice within an integrated multidisciplinary approach involving diabetologists, psychologists, and nutritionists [29,36]. Routine screening for depression and anxiety should therefore be incorporated alongside the assessment of DEBs in order to inform integrated, multidisciplinary care [37]. IIO is not merely a problem of treatment adherence, but rather reflects a complex interplay among weight control, body image, and the meaning attributed to glycaemia and insulin therapy [21]. Psychological support should therefore be central to the treatment of individuals with Diabulimia, particularly intervention targeting trauma and maladaptive beliefs about weight and appearance [29]. Treatment non-adherence is highly prevalent and determined by a combination of behavioural, economic, and organisational factors. Addressing it effectively requires enhanced patient education, sustainable and individualised prescriptions, and continuous attention to daily therapy management, recognising the central role of healthcare professionals in strengthening diabetes self-management skills [19]. In this direction, clinicians should explicitly explore missed injections, addressing issues such as pain, embarrassment, and regimen complexity, to adapt therapy to patients’ real lives and improve adherence and metabolic control [20]. In parallel, diabetes care teams must openly address weight-related concerns and fears associated with insulin therapy, working in an integrated manner with educational, dietary, and psychological professionals—particularly those with expertise in ED—to modify dysfunctional beliefs such as the association “insulin = weight gain” [21]. The most effective interventions should include work on pre-meal anxiety and guilt, reduction of rigid and punitive approaches to food and diabetes management, treatment of diabetes distress, and the use of targeted, “in-the-moment” interventions rather than exclusively generic therapies [24]. Finally, Coleman and Caswell [29] highlight the importance of protective factors in the recovery process, including supportive relationships with family, partners, peers, and online communities; psychological support; personal motivations related to life goals and prevention of future complications; and increased awareness and training for both healthcare professionals and patients.

## 5. Limitations and Future Directions

Despite the growing attention to the phenomenon of diabulimia, the available scientific evidence remains limited. The existing literature is characterised by a predominance of cross-sectional studies, a limited number of longitudinal investigations, and marked methodological heterogeneity. Moreover, diabulimia represents a hybrid clinical condition at the intersection of metabolic disorders and ED, which poses substantial challenges for integrated care pathways between diabetes care services and mental health services. These challenges are further compounded by the limitations of current diagnostic definitions and by the absence of formal recognition in major diagnostic manuals (DSM-5 [47,48], ICD-11 [49]), with important implications for case identification, research comparability, and treatment development. Another issue awaiting settlement is whether IIO is more frequent in one gender, similarly to the eating and feeding disorders that are more prevalent in women. Although a meta-analysis showed a higher prevalence in female patients with T1D [50], the fact that many studies included women-only populations may render this result doubtful. Another issue needing clarification is DSED. This entity is not strictly IIO and has not received due attention. The link between eating disorders and diabetes is intricate and complex [51], and DSED was found not to be exclusively linked to T1D but rather more frequent in T2D [52]. Given these limitations, the literature highlights the need for shared diagnostic criteria and better integration between diabetes services and mental health care to improve early identification and management before chronic complications develop [21,28,29]. From a perspective of early screening, the identification of a metabolic signature of IIO represents a promising avenue for future research. In this context, Pinhas-Hamiel et al. [23] proposed a clinical algorithm based on longitudinal analysis of HbA1c levels and the application of machine learning techniques. The identified metabolic pattern, observed more frequently in adolescent females, is characterised by persistently elevated values, high glycaemic variability, and abrupt deteriorations over time. These findings suggest that the use of objective metabolic indicators could form the basis for earlier and more targeted screening tools, with potential positive implications for clinical interventions. Future research may also explore the role of gut microbiota alterations as potential biological biomarkers of DSED and IIO, given emerging evidence linking unfavourable microbial profiles and reduced short-chain fatty acid-producing taxa to metabolic deterioration in this population [32].

## 6. Conclusions

Diabulimia is a complex condition that lies at the intersection of metabolic disease and eating disorder psychopathology and cannot be reduced to simple treatment non-compliance. Despite a high level of awareness of medical risks, the persistence of insulin omission indicates that this behaviour is driven by underlying psychological mechanisms rather than by a deficit in knowledge, thereby requiring integrated, multidisciplinary interventions that extend beyond diabetes education alone [29]. Diabulimia appears to function as a maladaptive coping strategy aimed at weight control, emotional regulation, and the restoration of autonomy in the context of a demanding chronic illness. Effective management therefore requires close collaboration between diabetes services and mental health services, with clinical attention focused on eating disorder psychopathology; body image disturbances; difficulties in accepting diabetes, particularly in younger patients; and the possible role of trauma and self-harming processes. The absence of formal diagnostic recognition continues to limit early identification and the development of structured care pathways. Recognising diabulimia as a distinct, hybrid clinical entity is essential to improving detection, treatment, and long-term outcomes.

## Figures and Tables

**Table 1:** Summary of included studies in chronological order.

Study	Site(s)	Population	Design	Results	Conclusions/Observations
Polonsky et al., 1994 [13]	1, Joslin Diabetes Center, Boston, Massachusetts, USA	381 ♀ with IDDM 13–60 yr-old, $\bar{x}$ age = 33.1 ± 12.4	Cross-sectional; survey completion about insulin dose omission; evaluation of frequency	104 (30.5%) pts regularly omitted doses	IDDM pts often skip insulin doses (1 out of 3). Reasons not investigated
Khan & Montgomery, 1996 [14]	5, 1, Manchester Diabetes Centre, Manchester, UK 2, Sheffield Diabetes Centre, Sheffield, UK 3, Lister Hospital, Stevenage, Hertfordshire, UK 4, Newham General Hospital, London, UK 5, Florence Diabetic Unit, East London, UK	48 ♀ YFDs 13–20 yr-old, $\bar{x}$ age = 17.02; 48 ♀ HC, $\bar{x}$ age = 17.02	Comparative observational study, cross-sectional design. Psychological Assessment: EDI, DEBQ, Insulin Questionnaire	EDI: YFDs scored ↑ ($\bar{x}$: 3.31, SEM: 0.55) than controls (1.67 ± 0.34) on each of the primary components of ED: DT (*F*_(1,88)_ = 60.1, *p* < 0.001), BD (*F*_(1,88)_ = 9.18, *p* < 0.005) and BN (*F*_(1,88)_ = 6.47, *p* < 0.002). The secondary components of the EDI (ineffectiveness, perfectionism, interpersonal distrust, interoceptive awareness and maturity fears) failed to reveal any significant effects (all *p* values > 0.05). DEBQ: YFDs had ↓ scores (2.82 ± 0.11) than controls (3.10 ± 0.08), *F*_(1,88)_ = 4.16, *p* < 0.05). Insulin Questionnaire: 22 (45.8%) YFDs were aware of the relationship between insulin and weight, and 11 (22.9%) were currently or had been IIO more than once a month. EDI, DEBQ, Insulin Questionnaire: There were no differences between those who understood the association between insulin and weight gain and those who did not (all *p* > 0.10). Those who IIO had ↑ scores for BN, ineffectiveness, interoceptive awareness and external eating (all *p* < 0.05), and ↓ scores on the DT scale (*p* < 0.001)	YFDs had ↑ scores for the three primary components of the EDI (DT, BN and BD). Those who had IIO had ↑ scores for ineffectiveness and interoceptive awareness and had ↑ sensitivity to external eating cues. Dieting and weight gain associated with diabetes treatment could precipitate a deterioration in eating attitudes. Omission of II and ↑ sensitivity to external eating cues may serve as markers for a group of YFDs who are at ↑ risk of developing an ED
Takii et al., 1999 [15]	>3, 1, Department of Psychosomatic Medicine, Graduate School of Medical Sciences, Kyushu University, Fukuoka, Japan 2, Diabetes Center at Tokyo Women’s Medical College, Tokyo, Japan 3, Other hospitals (not specified)	22 ♀ YFD + BN: $\bar{x}$ age = 23.2 ± 4.4; 11 ♀ YFD + BED: $\bar{x}$ age = 24.8 ± 7.5	Cross-sectional design that compares two groups: pts with T1D and BN with severe insulin omission and pts with T1D and BN without severe insulin omission. HbA1c level	ICB in BN group: Severe insulin omission: *n* = 16 (72.7%); Severe insulin omission alone: *n* = 12 (54.5%); With self-induced vomiting: *n* = 3 (13.6%); With excessive exercise: *n* = 1 (4.5%); Without Severe insulin omission: *n* = 6 (27.3%); Self-induced vomiting and misuse of laxatives: *n* = 2 (9.1%); Self-induced vomiting alone: *n* = 2 (9.1%); excessive exercise alone: *n* = 1 (4.5%); Fasting alone: *n* = 1 (4.5%); BN patients with “severe insulin omission” (*n* = 16) had significantly higher HbA1c levels ($\bar{x}$ ± SD, %) than those without (*n* = 6): 13.1 ± 2.6 versus 10.1 ± 0.8 [*F*_(1,20)_ = 57.10, *p* < 0.05]. Multiple regression analysis highlighted that “Severe insulin omission” (*p* < 0.001) was found to be most predictive of higher HbA1c levels in T1D ♀ with binge eating (BN + BED)	Severe insulin omission in women with T1D and BN is associated with significantly higher HbA1c, indicating extremely poor metabolic control due to prioritisation of weight control over glycaemic management
Goebel-Fabbri et al., 2008 [16]	1, Behavioral and Mental Health Research, Joslin Diabetes Center, Boston, Massachusetts, US	234 ♀ with T1D, $\bar{x}$ age at F-U was 45 ± 12 years (range 24–72)	Longitudinal observational study; BL: demographic and clinical information (age, diabetes duration, BMI, medical complications and HbA1C); F-U: self-report questionnaire assessing age, diabetes duration, BMI, and medical complications. Psychosocial assessment: Self-administered survey about insulin restriction, SCI for diabetes, PAID for diabetes-specific distress, HFS, BSI, BULIT-R and EDI	71 (30%) pts reported insulin restriction at BL. BL differences: Insulin restrictors were younger (32 vs. 36 years, *p* < 0.01); had higher HbA1c levels (9.6 vs. 8.3%, *p* < 0.001); no differences in BMI or diabetes duration; reported poorer diabetes self-care (SCI: 50 vs. 70.4, *p* < 0.001), higher distress (PAID: 69 vs. 34.5, *p* < 0.001); higher fear of hypoglycaemia (HFS: 35.1 vs. 26.5, *p* < 0.01); more psychological symptoms (BSI: 60.4 vs. 54.4, *p* < 0.001); and more eating disorder symptoms (BULIT-R: 66.8 vs. 45.6; EDI: 37.9 vs. 22.3; *p* < 0.001). Diabetes complications (F-U): ↑ rates of nephropathy (25% vs. 10%, *p* < 0.01). ↑ rates of foot problems (25% vs. 12%, *p* < 0.05). No significant differences in retinopathy, neuropathy, or cardiovascular complications. IIR increased risk of death by × 3.2 during the 11-yr F-U	Insulin restriction is associated with increased mortality; restrictors died younger (45 vs. 58 years) and had higher rates of nephropathy and foot problems. Mortality mainly related to eating disorder symptoms and diabetes-specific distress
Takii et al., 2008 [17]	2, 1, Department of Psychosomatic Medicine, Graduate School of Medical Sciences, Kyushu, JP 2, Tokyo Women’s Medical University, JP	109 ♀ YFDs (BN = 70, BED = 28, AN = 7, ED-NOS = 4); Retinopathy group: None: $\bar{x}$ age = 22.9 ± 5.2 Simple retinopathy: $\bar{x}$ age = 24.3 ± 2.4; Advanced retinopathy: $\bar{x}$ age = 27.6 ± 5.1 Nephropathy group: Absent: $\bar{x}$ age = 23.3 ± 5.0 Present: $\bar{x}$ age = 27.0 ± 4.7	Observational study, cross-sectional design. Clinical assessment: HbA1c level, fundus examination; microalbuminuria.	Retinopathy and nephropathy were significantly associated with the duration of severe IIO and with the duration of DM-1. Retinopathy: OR = 1.35 (insulin) and 1.23 (diabetes) Nephropathy: OR = 1.35 (insulin) and 1.21 (diabetes). No significant association with HbA1c at first visit or with other compensatory behaviours (vomiting, laxative use).	Prolonged IIO leads to extremely poor glycaemic control and ↑ the risk of complications. The authors recommend that patients be informed early about these risks and that targeted interventions be implemented
Ackard et al., 2008 [18]	1, Children’s Hospital in St Paul, MN, USA and a population—based school sample.	143 adolescents with T1D + adolescents in comparison group; T1D group: ♂ 73 and ♀ 70; Only T1D included. General adolescent population: 2377 males and 2357 females (non-diabetic sample)	Cross-sectional comparative study; survey on demographic characteristics and mean BMI.	Overall, adolescents with type 1 diabetes showed: Lower levels of weight dissatisfaction; Less engagement in unhealthy weight control behaviours (dieting, fasting); More regular meal patterns. Gender differences were noted: Females with type 1 diabetes were less likely to diet, fast, or severely restrict food compared to their peers without diabetes. Males with type 1 diabetes were less likely to engage in healthy weight-control strategies like exercising or eating more fruits and vegetables. A worrisome finding was the use of insulin manipulation as a weight-control method: 1.4% of ♂ and 10.3% of ♀ reported IIO; 1.4% of ♂ and 7.4% of ♀ reported IIR	Adolescents with type 1 diabetes, despite receiving medical supervision, still demonstrate unhealthy weight-control behaviours. The practice of insulin omission or reduction for weight loss is especially dangerous, as it can lead to severe complications. The study emphasises the necessity of ongoing psychological and nutritional support for young people managing T1D
Adisa et al., 2009 [19]	1, Secondary healthcare facility in Ibadan, Southwestern Nigeria	121 pts with T2D; 60 ♂ (49.6%), 61 ♀ (50.4%)	Cross-sectional design; survey on socio-demographic characteristics, medication nonadherence, self-management behaviours, and diabetes knowledge	85 patients (70.2%) reported dose omission; main reasons included dissatisfaction with daily medication intake (*n* = 54, 44.6%), inconvenience of taking medication outside home (*n* = 24, 19.8%), fear of taking too many drugs at a time (*n* = 10, 8.3%), busy work schedule (*n* = 14, 11.6%), unpleasant taste (*n* = 8, 6.6%)	Pts with T2D skip insulin doses principally for dissatisfaction with daily medication intake and inconvenience of taking medication outside home
Peyrot et al., 2010 [20]	1. Loyola University Maryland-Johns Hopkins, Baltimore, MD; 2. Henry Ford Health Systems, Detroit, MI; 3. Univ. of Texas Med Branch, Galveston, TX, USA	502 adults with T1D or T2D who take insulin (276 ♂, 226 ♀); $\bar{x}$ age = 54.9 yrs; SD = 13.9 yrs	Cross-sectional design, based on an internet survey. Clinical and psychological assessment: basic demographic information; disease type, duration, complications, and treatment; perceived burden of II; the experience of II; negative affect toward II, frequency of skipping II	57% reported skipping II, 20% reported skipping them sometimes or often. Those who said that taking injections interfered with activities of daily living intentionally skipped II more often.	IIO occurs in the majority of adults using insulin to treat their diabetes (20%) and varies with a number of demographic and disease characteristics. The authors acknowledge DIA as a phenomenon reported in previous studies among ♀ adolescents, but in their sample, they found no evidence of IIO related to weight control
Goebel-Fabbri et al., 2011 [21]	1, Joslin Diabetes Center, Boston, Massachusetts, USA	207 adult ♀ with T1D, originally recruited from a previous study. $\bar{x}$ age 45 yrs; mean diabetes duration 28 yrs.	11-year longitudinal study. The research examined patterns of insulin restriction (intentional reduction or omission of insulin for weight control): whether ♀ continued, stopped, or newly developed this behaviour over time. Measures included HbA1c, diabetes complications, quality of life, and mortality	Insulin restriction was not stable over time → some ♀ improved (stopped restricting) → others newly began restricting during F-U. ♀ who restricted insulin showed: Higher HbA1c levels; More diabetes-related complications; Worse psychological health and quality of life. Higher mortality rates among ♀ who continued or began insulin restriction	Insulin restriction can emerge later during type 1 diabetes, not only during adolescence. The behaviour is also strongly linked with serious medical risks, including increased likelihood of death. Ongoing monitoring of insulin use is essential in clinical care. Psychological and nutritional support should be provided to prevent and address these behaviours
Wisting et al., 2013 [22]	1, Norwegian Childhood Diabetes Registry, Norway	770 pts with T1D, 11–19 yr-old, $\bar{x}$ age = 14.6 ± 2.1; 380 ♂ (49.4%), 390 ♀ (50.6%)	Cross-sectional epidemiological survey of the nationwide; DEPS-R, EAT-12	243 (31.6%) used less insulin; 53 (6.9%) skipped insulin; ♀: 144 (36.8%) restricted, 102 (26.2%) skipped; ♂: 36 (9.4%) restricted, 17 (4.5%) skipped. Insulin restrictors were significantly older ($\bar{x}$: 15.0, SD: 2.0 vs. 14.4 ± 2.1; *p* < 0.001), had significantly ↑ HbA1c (9.0 ± 1.7 vs. 8.3± 1.2; *p* < 0.001), and had significantly ↑ scores on both the DEPS-R (19.3 [SD, 12.2] vs. 6.8 [SD, 6.8]; *p* < 0.001) as well as the EAT-12 (3.0 [SD, 3.2] vs. 1.4 [SD, 2.2]; *p* < 0.001) than non-restrictors	31.6% of pts admitted to restricting insulin at least on an occasional basis after overeating. Pts ↓ or omit insulin for fear of hypoglycaemia, interference with activities of daily living, injection pain, injection embarrassment, negative affect toward injections or for weight control purposes. Nevertheless, the clinical effects of insulin restriction are detrimental on physiology and are important to the pathogenesis of short- and long-term consequences. Poor metabolic control may place individuals at risk of ↑ morbidity and mortality
Pinhas-Hamiel et al., 2013 [23]	1, Maccabi Juvenile Diabetes Center, Tel Aviv, Israel	287 pts (132 ♀; 155 ♂) with T1D and 26 ♀ with T1D and IIO	Cross-sectional; Observational and retrospective; Clinical assessment: HbA1c level	Adolescents with IIO were discriminated by female sex, HbA1c levels greater than 9.2%, more than 20% of HbA1c measurements above the 90th percentile for age, a mean of the three highest HbA1c delta z-scores greater than 1.28, current age of 13.7 yrs or older, and age at diagnosis of 13.8 yrs or older	The coexistence of diabetes and IIO is associated with poor glycaemic control and a high risk of acute and chronic complications. The age of onset of DM-1 and shorter disease duration were reported to be associated with excess risk of subsequent development of severe ED in ♀ pts
Merwin et al., 2015 [24]	1, Duke University Medical Center, Durham, North Carolina and collaborating sites, USA	83 adults with T1D exhibiting eating disorder symptoms ($\bar{x}$ age: 41.89; ♀: 88%; Mean diabetes duration: 23.43 yrs)	Cross-sectional; Ecological Momentary Assessment (EMA) over three days, with multiple real-time measurements via smartphone. Assessed: Episodes of insulin restriction; Emotional states (anxiety, sadness); Weight/shape concerns; Interpersonal experiences; Binge eating episodes	The participants reported at least one insulin restriction episode during the three-day period. Episodes were more likely when: Weight/shape concerns ↑; Negative emotions intensified: Anxiety; Sadness; Same day binge eating occurred; Negative emotions often preceded insulin restriction episodes	Insulin restriction is triggered by short-term emotional and cognitive factors related to body image and psychological distress. Behaviour can be impulsive and reactive, not solely tied to long-term weight goals. Interventions should target: Emotional regulation, Management of weight-related thoughts, Prevention of binge episodes. Real-time monitoring may help identify immediate risk and enable timely intervention
Bächle et al., 2016 [25]	1, German Center for Diabetes Research (DZD), Düsseldorf, Germany	819 pts with T1D, 11–21 yr-old, $\bar{x}$ age = 16.3 ± 2.3; 414 ♂ (50.5%), 405 ♀ (49.5%); Onset at the age of 0–4 yrs, $\bar{x}$ age = 3.0 ± 1.2; Diabetes duration almost 10 yr, $\bar{x}$ age = 13.3 ± 2.0	Comparative Cross-sectional design; SCOFF questionnaire for ED; Evaluation of frequency insulin restriction (i.e., >5 ×/wk); N° of carbohydrate exchange units consumed without insulin; HbA1c level; N° of in-pt-treated DKA during the last 12 months	60 pts (7.1%) demonstrated insulin restriction; Pts with insulin restriction omitted insulin injections × ≥2 the No. of carbohydrate exchange units compared with pts without insulin restriction; HbA1c values were highest (*p* < 0.05) in pts with frequent insulin restriction (8.9–10.0%) compared to pts without insulin restriction (8.1–8.7%). ♀ pts with insulin restriction and a positive ED screening showed higher DKA incidence (27.3 per 100 person-years) compared with ♀ pts without insulin restriction but with a positive ED screening (16.7) or ♀ pts without insulin restriction and ED screening (8.4)	Youth pts with insulin restriction showed an increased risk of diabetes-related complications, as corroborated by the partly increased rate of inpatient-treated diabetic ketoacidosis. In particular, female sex with the combination of insulin omission and coexisting disordered eating behaviours substantially increases the risk of acute metabolic complications
Wisting et al., 2019 [26]	1, Norwegian Childhood Diabetes Registry, Norway	104 adolescents with T1D (58.1% ♀, 41.9% ♂), $\bar{x}$ age = 15.7, SD = 1.8, range 12–20 yrs, age at T1D onset = 9.6 yrs, Mean T1D duration = 5.6 yrs, mean zBMI = 0.4	Cross-sectional and observational design. Psychological Assessment: Child Eating Disorder Examination (ChEDE)	In ♀ adolescents with DM-1, eating meals irregularly or too infrequently was linked to worse metabolic outcomes and ↑ levels of ED symptoms, such as binge eating, self-induced vomiting, and IIO. In contrast, among ♂ adolescents with DM-1, meal frequency and eating patterns were related only to attitudinal aspects of ED pathology, not to EDB or metabolic control	Evaluating eating habits is a simple and practical indicator that can be easily integrated into routine DM-1 management. When meal patterns are irregular or infrequent, this should prompt a more thorough assessment for ED behaviours, including IIO
Papadakis et al., 2019 [27]	1, Children’s Hospital sites in the USA	54 youth with T1D ages 12–17 ($\bar{x}$ age = 14.7, 55.6% ♀)	Cross-sectional; youth and caregivers completed an annual psychosocial screening battery that included the PROMIS^®^ depression and anxiety scales, the Problem Areas in Diabetes measure, and the Diabetes Family Conflict Scale	Intentional omission of insulin to lose or avoid gaining weight was significantly positively correlated with youth report of depression, anxiety, diabetes distress, and diabetes-related family conflict. Compared to youth who do not intentionally omit insulin, those who do omit insulin were more likely to have clinically elevated levels of depression (*p*’s < 0.05). No differences in parent-reported psychosocial outcomes	Results reveal that youth who intentionally omit insulin for weight purposes are at risk of poorer psychosocial outcomes, especially depression. Additional research is needed to understand the causal direction of these associations, and to examine the potential role of disordered eating behaviour. Results also highlight how psychosocial screening during routine diabetes visits can identify youth and caregivers in need of behavioural health interventions
Luyckx et al., 2019 [28]	1, Belgian Diabetes Registry	300 pts with T1D, 16–28 yr-olds, $\bar{x}$ age = 20.80 ± 3.31; 130 ♂ (43.2%), 170 ♀ (56.7%)	Longitudinal observational study; DEPS-R for the evaluation of insulin restriction or omission, SCI for diabetes, PAID for diabetes-specific distress, HbA1c for blood glucose level, T1 to T4 were evaluation points at a yr of distance each	At T1, 46 (15.3%) pts reported restricting insulin at least sometimes, and 13 (4.3%) pts reported omitting insulin at least sometimes; at T2, 31 (10.3%) pts and 11 (3.7%) pts reported insulin restriction or omission, respectively. There were no sex differences in insulin restriction or omission T1–T2 [*x^2^*(2) = 1.524–5.062; *p* = 0.080–0.467]. Considering ED subgroups, at T1, 33–38 (31.8–36.8%) pts with ED reported restricting insulin at least sometimes, compared to 16 (8.1%) pts without ED; for insulin omission, the numbers were 11–19 (10.5–18.2%) pts versus 1 (0.5%) pt. At T2, 24–34 (22.8–33.3%) pts with ED reported restricting insulin at least sometimes, compared to 8 (4.1%) pts without ED; for insulin omission, the numbers were 9–21 (8.8–20.8%) pts vs. 1 (0.5%) pt	The high occurrence of insulin restriction and omission found in the current study highlights the need for explicit attention to ED in youth pts. Reasons probably include more weight concern and body image dissatisfaction
Coleman & Caswell, 2020 [29]	1, University of Central Lancashire, UK	45 pts with T1D (42 ♀, 2 ♂, 1 not specified), $\bar{x}$ age = 32.09, SD = 11.07, range = 15–58 yrs. $\bar{x}$ age of first restricting insulin = 19.31 (7.70).	Cross-sectional; Qualitative research design. Psychological assessment: EDE-Q; 16 open-ended questions to explore participants’ personal experiences and perspectives on insulin restriction.	38% reported frequently manipulating their insulin, 33% said they no longer do so, 27% did it occasionally, and 2% only rarely. 78% reported that their insulin restriction was mainly driven by wanting to lose weight; 18% explained that their primary motivation for limiting insulin was a strong dislike of diabetes and a desire to regain a sense of control; 4% reported that their primary motivation for misusing insulin was self-harm; 10 pts said weight loss was not their primary motive for restricting insulin but noted that it still played a secondary role; 73% reported having required medical or psychiatric treatment as a result of their insulin misuse. 87% said they were aware of the severe consequences of DIA yet still felt unable to stop limiting their insulin	Psychological care needs to be a central component of treatment for individuals with DIA. Clinicians must understand the physical and psychological challenges linked to insulin restriction, including concerns about weight and appearance, difficulties in adjusting to diabetes, past traumatic experiences, and the crucial role of supportive relationships
Beam et al., 2021 [30]	2, 1. Psychological Sciences and the Health Sciences Research Institute, University of California; 2. Department of Psychology, University of Utah, US	236 participants; High-school seniors (61% ♀) aged 17–18 years (M 1⁄4 17.7, SD 1⁄4 0.40)	Cross-sectional correlational design; HbA1c level, DERS, CES-D	IR and IRWC were not significantly associated with each other. IR was associated with self-management behaviours but not HbA1c, whereas the opposite was true for IRWC. All DERS subscales (M 1⁄4 10.60–16.73) and CES-D (M 1⁄4 16.56) were correlated with greater IRWC; CES-D and all but one DERS subscale were correlated with IR	This study demonstrated that emotion dysregulation and depressive symptoms are important correlates of the dangerous behaviour of IR, but function differently when insulin is restricted specifically for weight control versus unspecified reasons. Depressive symptoms played a central role in both forms of IR, accounting for the associations of emotion dysregulation with IRWC, and moderating the associations of multiple facets of emotion dysregulation with general IR
Şahin-Bodur et al., 2021 [31]	1, Ankara University Faculty of Medicine, Cebeci Hospital, Pediatric Endocrine Policlinic, Ankara, Türkiye	110 pts with T1D, 10–19 yr-old, $\bar{x}$ age = 14.0 ± 2.40; 57 ♂ (51.8%), 53 ♀ (48.2%)	Cross-sectional study; DEPS-R for the evaluation of insulin restriction or omission; HEI-2015 used to measure the diet quality of pts; HbA1c level	35 (31.8%) pts were found to be at risk of DIA. No significant difference was found between the genders (17 ♂ vs. 18 ♀, 48.6% vs. 51.4, *p* = 0.397). Pts with risk of DIA showed a significant (*p* < 0.05) lower total scores in HEI-2015 (63.3 ± 9.17) than those without risk (67.2 ± 9.50). High HbA1c levels were significantly more frequent in patients with DIA risk compared to those without DIA risk (100% vs. 86.8%, *p* < 0.05)	31.8% of pts were found to be at risk of DIA. Adolescents with TD1 at risk of DIA had lower diet quality. Elevated HbA1c levels may contribute to disordered eating behaviours, poor glycaemic control, and an increased risk of long-term complications, including cardiovascular events
Tarçın et al., 2023 [5]	1, Cerrahpaşa Faculty of Medicine, İstanbul, Türkiye	92 adolescents with T1D (45 ♂, 47 ♀), 12–18 yr-olds, $\bar{x}$ age = 15.5 ± 2.8 yrs, $\bar{x}$ duration of diabetes = 6.3 ± 3.9 yrs, mean HbA1c = 8.26 ± 1.50%	Cross-sectional study. Clinical and Psychological Assessment: Diabetes Eating Problem Survey-Revised (DEPS-R); to determine the risk of DSED. Eating Disorder Examination Questionnaire (EDE-Q); Revised child anxiety and depression scale (RCADS); Parenting Style Scale (PSS)	The DEPS-R flagged DSED risk in 23.9% (*n* = 22 youths, M/F = 9/13). DEPS-R scores were found to be significantly ↑ in the subgroup with an HbA1c above 9% (*n* = 34) than those with <9% (*n* = 58). Pts with divorced parents (10.9%) had significantly ↑ DEPS-R scores than those with married parents. EDE-Q scores were ↑ in the DEPS-R+ group compared with the DEPS-R− group (*p* = 0.006). RCADS mean scores were also significantly ↑ in adolescents at risk of DSED. PSS results showed that adolescents in the DEPS-R+ group had markedly ↓ psychological autonomy scores. 30 participants (32.6%) had one or more psychiatric diagnoses, and 2 were identified as having an ED. Gender was not a determinant	Implementing DEPS-R screening as part of routine care for adolescents with DM-1 may help detect DSED at an early stage. Prompt referral of ↑ risk pts to child psychiatry can ensure early management of ED and co-occurring psychopathologies
Igudesman et al., 2023 [32]	1, University of North Carolina at Chapel Hill (UNC) and Stanford University (US)	45 young adults with T1D aged 19–30 years; (T1D duration >1 year); ♀ 31, (68.9%)	Longitudinal study; Participants completed four measurement visits at BL and after three 3-month dietary periods. DEPS-R, HbA1c level and BMI index were used.	In young adults with T1D and overweight or obesity at BL who participated in a weight management trial, statistically significant inverse associations of DE (DEPS-R score and insulin restriction “At least sometimes” compared to “Rarely or Never”) with the normalised abundance of two SCFA-producing genera (*Anaerostipes* and *Lachnospira*) were found. Increased self-reported DE scores among individuals with T1D are consistently associated with elevated HbA1c and BMI, so it is possible that DE may further precipitate inflammation by contributing to ↑ hyperglycaemia and body weight. These hypothesised mechanisms may compound the inflammatory nature of autoimmunity in T1D, which itself has been linked with an altered composition of the intestinal microbiota and gut permeability	In young adults with T1D and overweight or obesity, it was found that increased levels of subthreshold DE and insulin restriction were associated with a reduced normalised abundance of SCFA-producing commensal microorganisms that may also be reduced in the nutrient-poor setting of anorexia nervosa and in the inflammatory state of T2D but that may have metabolic benefits for glycaemia. If reducing insulin restriction and other DE behaviours can promote replenishment of SCFA producers, which may in turn benefit metabolism, this is another reason to prioritise DE screening and intervention in T1D
Yafei et al., 2023 [33]	1, Jazan Endocrinology and Diabetes Centre (JEDC), Jazan, Saudi Arabia	265 pts with T1D, 12–25 yr-olds, $\bar{x}$ age = 17.7 ± 3.5; 102 ♂ (38.5%), 163 ♀ (61.5%)	Cross-sectional; DEPS-R for the evaluation of DEB and insulin restriction or omission. HbA1c level	73 (27.5%) pts restricted their insulin doses, and 32 (12.1%) skipped their insulin doses at least occasionally after overeating. Insulin restrictors had significantly higher HbA1c (9.2 ± 2.2 vs. 8 ± 2; *p* < 0.001), later onset of diabetes (11.6 ± 4.5 vs. 10.3 ± 4.2; *p* = 0.041), and significantly higher DEPS-R scores (24.8 ± 10.4 vs. 11.8 ± 8.4; *p* < 0.001) compared to non-restrictors. There was no statistically significant difference in insulin restriction or omission across genders. 29.4% of ♀ and 24.5% of ♂ reported restricting insulin when overeating, and 13.5% of ♀ skipped their insulin doses at least occasionally, compared to 9.8% of ♂ (*p* > 0.05 in both)	27.5% of pts demonstrate insulin restriction and another 12.1% skip insulin dose entirely at least occasionally after overeating. Reasons are different, including weight loss. Adolescents and young adults who restrict insulin have higher HbA1c levels and higher DEPS-R scores. Poor glycaemic control is linked to an increased incidence of DKA and other metabolic or vascular complications of diabetes
Ip et al., 2023 [34]	1, Touro University California, USA	225 adults with T1D (178 ♀, 45 ♂, 2 not specified); without DIA (*n* = 205): $\bar{x}$ age = 34.5 ± 11.4 yrs; with DIA (*n* = 20), $\bar{x}$ age = 30.9 ± 10.9 yrs. $\bar{x}$ age onset of DIA = 17.5 ± 7.9 yrs	Cross-sectional study; Web-based survey with the aim of estimating the prevalence of DIA.	DIA was identified in 8.9% (20/225) of adult pts with DM-1. Pts who were identified as having DIA exhibited a ↑ rate of diagnosed MDD than did pts without it (40% vs. 11.5%) and had ↑ HbA1c levels (8.4% vs. 6.9%). They demonstrated ↑rates of hospital visits or hospitalisation for diabetes-related emergencies in the past 12 months than did pts without it (30.0% vs. 13.2%). There was no significant difference in DIA rates between ♂ and ♀ (4.5% vs. 10.0%). The majority of pts were ♀ (90.0%). More than half did not inform their healthcare provider that they were IIO for weight-loss purposes (65.0%). Most pts with DIA (80%) were not familiar with the DIA helpline	Pts identified with DIA showed ↑ HbA1c levels, were more likely to have diabetes-related emergency department visits or hospitalisations and had a greater prevalence of MDD compared with those without DIA. ↑ HbA1c levels values and the presence of MDD were the key factors linked to DIA
Chou et al., 2023 [35]	1, Taiwan, China	142 Chinese pts diagnosed with T1D (*n* = 110) and T2D (*n* = 32) aged 10–30 years; ♂ T1D: 50 (45.5%); ♂ T2D: 16 (50%); ♀ T1D: 60 (54.5%); ♀ T2D: 16 (50%)	Cross-sectional study; (TFEQ-R21) and the modified SCOFF (mSCOFF) questionnaire were used for the evaluation of DE/IR behaviour. HAD anxiety and depression were also used	17.6% pts restricted insulin use and 6.3% self-medicating for weight control (higher in T2D than T1D)	Patients with T2D had a greater concern for body image and were more likely to use weight-control medications or restrict insulin use than those with T1D. Stratifying pts by weight status, a trend for pts who had overweight/obesity to display ↑ DE/IR behaviours was found. DE/IR behaviour was associated with psychological distress, i.e., anxiety and depression based on the HAD scale. Significant association between DE/IR behaviour and HbA1c-SD, extending evidence that DE/IR behaviour may have clinical relevance to stability in glycaemic excursion in these patients
Hartlaub & Hill, 2025 [36]	1, West Chester University, Pennsylvania, USA	199 adults with T1D (87 ♂, 110 ♀, non-binary = 2); $\bar{x}$ age = 29,23 yrs; SD = 5,67; range 18–40 yrs	Cross-sectional observational study; Survey online (Qualtrics); Eating Disorder Examination Questionnaire (EDE-Q); DEPS-R; The Diabetes Distress Scale (DDS); The Physical Appearance Comparison Scale—Revised (PAC); Body Esteem Scale for Adolescents and Adults (BESAA)	Pts who had received an ED diagnosis were mostly diagnosed with BED (*n* = 20; 10%) and AN (*n* = 17; 8.5%). Diabetes distress showed the strongest link to both DEB and the IIO. The majority of pts (131, 65.8%) said they spent 1–5 h for day on social media, and the platforms they used most often were those centred on images—such as Instagram (142 pts, 71%). The study found that how participants viewed BMI was not significantly related to IIO, dietary restraint, or DEB. The sample showed a ↑ prevalence of diagnosed ED (22%), as well as a high proportion of pts reporting DEB (scores ≥ 20 on the DEPS-R; 85 pts, 43%). Overall mental health was reported as fair to poor (40.7% of the sample)	The authors found a significant relationship between diabetes distress and all three outcomes: DEB, dietary restraint, and IIO. In addition, body-image-related factors such as weight esteem and physical appearance comparisons on social media were both significantly associated with DEB and dietary restraint
Oikonomou et al., 2025 [4]	3, Salonica, Aristotelion, AHEPA and Hippokration, Greece	120 Greek pts (61 ♂, and 59 ♀, aged 9–18 years) diagnosed with T1D at least 1 year before the start of the study	Cross-sectional observational study; DEPS-R questionnaire for the evaluation of the risk of DEBs specifically in patients with T1D and was used to assess disordered eating tendencies and explore their relationship with demographic, anthropometric, and glycaemic parameters	Girls, compared with boys, scored significantly higher on the DEPS-R. Significant positive associations were observed between the DEPS-R score and both age (*r* = 0.212, *p* = 0.020) and Body Mass Index (BMI) (*r* = 0.419, *p* < 0.001). A significant association with HbA1c (*r* = 0.182, *p* = 0.047) suggested that poorer glycaemic control may be linked to disordered eating	Female sex, older age, higher BMI, and elevated HbA1c were significantly associated with higher DEPS-R scores, suggesting heightened vulnerability to DEBs
Figueiredo et al., 2026 [37]	1, State University of Campinas, Campinas, Brazil	217 adolescents and young adults aged 13–39 yrs ($\bar{x}$ = 28.5 yrs, SD = 6.83; 22 ♂, 195 ♀) with confirmed T1D for ≥1 year	Cross-sectional; participants from all over Brazil completed online questionnaires, including DEPS-R and HAD, Brazilian versions	IIO found in 44 participants (20.3%), IIR in 139 (64.1%). ↑ DEPS-R scores independently associated with IIO/IIR, (*p* < 0.001), body comparison (*p* < 0.001), frequent family comments about body shape (*p* = 0.039), ↑ HAD depression (*p* < 0.001), BMI (*p* = 0.007), HbA1c (*p* = 0.001), ↓ income (*p* = 0.004), and being ♀ (*p* < 0.001)	One in five T1D patients skip insulin and >50% restrict it, with ♀ at ↑ risk and scoring ↑ on depression and diabetes-related eating problems. Social and family factors affect eating behaviour in T1D pts and have psychological consequences
Ritz et al., 2026 [38]	1. SFDT1 cohort database from France, investigators based in various locations (24 in France, 1 in US)	1113 participants with T1D, 568 ♂, 545 ♀, *μ*_½_ 38 (IQR 29–50) yrs, diabetes duration *μ*_½_ 21 (IQR 12–32) yrs	Cross-sectional online survey of adult pts with T1D using the SCOFF and one question regarding IIO	noED/noIIO: 68% (*n* = 758), ED/noIIO: 11% (*n* = 124), noED/IIO: 16% (*n* = 177), ED/IOM: 5% (*n* = 54). Compared with noED/noIIO, ED/noIIO, TIR (OR 0.5; 95% CI, 0.4 to 0.7) and TBR (OR 0.5; 95%CI 0.3 to 0.8) inversely associated with ED/IIO. TIR (OR 0.4; 95%CI 0.4 to 0.5) associated with noED/IIO. TAR (OR 2.2; 95%CI 1.6 to 2.9), GRI (OR 1.8; 95%CI 1.3 to 2.5), glucose monitoring indicator (OR 2.2; 95%CI 1.7 to 2.9) and HbA1c (OR 2.0; 95%CI 1.5 to 2.5) directly associated with ED/IIO	Both ED and IIO are frequent in T1D; IIO associated with impaired glycaemic control. Reason for skipping insulin injections not assessed. Associations remained in both genders
South et al., 2026 [2]	1. Canadian Registry (investigators based in Québec and Ontario Universities)	1194 participants with T1D responded; excluded were pregnant (*n* = 6), not on insulin (*n* = 2) and aged <18 yrs (*n* = 36); 1150 responded to the IIO question (765 ♀, 379 ♂, 6 not declared; age $\bar{x}$ = 45.3 ± 14.9)	Cross-sectional; Canadian T1D participants completed online questionnaires, including demographic data and lifestyle and mental health variables (only adults were analysed) and the PHQ-9	IIO Yes 84 (7.3%; 76 ♀, 8 ♂, age $\bar{x}$ = 39.6 ± 12.2) No 1066 (689 ♀, 377 ♂, age $\bar{x}$ = 45.7 ± 15.0; corrected *p* = 0.0012 for both gender and age). Multivariable logistic regression identified factors involved in IIO; being a ♀ [OR: 5.73, 95%CI: 2.65 to 14.98], having reported ↑ diabetes distress [OR:1.62, 95%CI: 1.32 to 2.00], and ↑ weight cycling [OR: 1.08 to 95%CI: 1.02, 1.13] → ↑ odds for IIO; ↑ age at diagnosis → ↓ odds for IIO. People with IIO scored higher on PHQ-9 depression (10.6 ± 6.4 vs. 6.1 ± 5.4; *p* < 0.001). Insufficient evidence of an association between BMI and IIO (1.04, 95%CI [0.99 to 1.08])	IIO is associated with adverse health outcomes (weight cycling, diabetes distress, depression) and should be regularly screened in pts with T1D

*Abbreviations:* AE(s), adverse events(s), side effect(s); AN, anorexia nervosa; BD, body dissatisfaction; BED, binge eating disorder; BESAA, Body Esteem Scale for Adolescents and Adults; BL, baseline; BMI, body mass index; BN, bulimia nervosa; BSI, Brief Symptom Inventory; BULIT-R, Bulimia Test–Revised; CES-D, Center for Epidemiologic Studies Depression scale; ChEDE, Child Eating Disorder Examination; DB, double-blind; plac, placebo; DDS, Diabetes Distress Scale; DEB, Disordered Eating Behaviour; DEBQ, Dutch Eating Behaviour Questionnaire; DEPS-R, Diabetes Eating Problem Survey-Revised; DERS, Difficulties in Emotion Regulation Scale; DIA, diabulimia; DKA, diabetic ketoacidosis; DM-1, type 1 diabetes mellitus; DM-2, type 2 diabetes mellitus; DSED, diabetes-specific eating disorder; DT, drive for thinness; EAT, Eating Attitudes Test; ED, eating disorders; EDE-Q = Eating Disorder Examination—Questionnaire; EDI, Eating Disorder Inventory; ED-NOS: eating disorder not other-wise specified; F-U, follow-up; GRI, Glycaemic Risk Index; HAD, Hospital Anxiety and Depression Scale; HbA1c, Serum glycosylated haemoglobin; HC, healthy controls; HEI-2015, Healthy Eating Index-2015; HFS, Hypoglycaemia Fear Survey; ICB, Inappropriate compensatory behaviours; IDDM, insulin-dependent diabetes mellitus; II, insulin injections; IIO, intentional insulin omission; IIR, intentional insulin restriction; IQR, interquartile range; MDD, Major Depressive Disorder; OR, odds ratio; PAC, The Physical Appearance Comparison Scale—Revised; PAID, Problem Areas in Diabetes; PHQ-9, Patient Health Questionnaire-9; PSS, Parenting Style Scale; pt(s), patient(s); RCT, randomised controlled trial; RCADS, Revised child anxiety and depression scale; SCI, Self-Care Inventory; SCOFF, Sick, Control, One, Fast, & Food; SD or ±, standard deviation; SEM, standard error of mean; SFDT1, French-Speaking Diabetes Society—Type 1 Diabetes Cohort; TAR, % time spent >180 mg/dL; TBR, % time spent <70 mg/dL; TIR, % of time spent in the range of 70 and 180 mg/dL; T1D, type 1 diabetes; T2D, type 2 diabetes; wk(s), week(s); YFDs, young females with diabetes; $\bar{x}$, mean; *μ*_½_, median; 95%CI, 95 percent confidence interval; ↓, decreased, diminished, drop, lower; ↑, increased, augmented, greater than, higher; →, leads to, follows, subsequently; ≈, about, approximately; ≠, different from; ♀, female; ♂, male.

**Table 2:** Risk of Bias assessment using the ROBINS-I V2 tool (including overall bias) for each study.

Study	Domain						Overall Bias
	D1	D2	D3	D4	D5	D6	
Polonsky et al., 1994 [13]	Low	Moderate	Low	Moderate	Low	Low	Moderate
Khan & Montgomery, 1996 [14]	Low	Moderate	Moderate	Low	Moderate	Moderate	Moderate
Takii et al., 1999 [15]	Low	Low	Moderate	Low	Low	Moderate	Moderate
Goebel-Fabbri et al., 2008 [16]	Low	Moderate	Low	Moderate	Low	Moderate	Moderate
Takii et al., 2008 [17]	Low	Moderate	Serious	Low	Low	Low	Serious
Ackard et al., 2008 [18]	Low	Moderate	Moderate	Low	Low	Low	Moderate
Adisa et al., 2009 [19]	Moderate	Moderate	Moderate	Moderate	Moderate	Moderate	Moderate
Peyrot et al., 2010 [20]	Low	Moderate	Serious	Low	Low	Moderate	Serious
Goebel-Fabbri et al., 2011 [21]	Low	Low	Low	Low	Low	Low	Low
Wisting et al., 2013 [22]	Low	Moderate	Low	Low	Low	Low	Moderate
Pinhas-Hamiel et al., 2013 [23]	Low	Moderate	Moderate	Low	Low	Low	Moderate
Merwin et al., 2015 [24]	Low	Moderate	Moderate	Low	Moderate	Low	Moderate
Bächle et al., 2016 [25]	Low	Moderate	Low	Low	Low	Low	Moderate
Wisting et al., 2019 [26]	Low	Low	Low	Low	Low	Low	Low
Papadakis et al., 2019 [27]	Moderate	Moderate	Low	Low	Low	Low	Moderate
Luyckx et al., 2019 [28]	Low	Moderate	Moderate	Moderate	Low	Low	Moderate
Coleman & Caswell, 2020 [29]	Serious	Moderate	Serious	Low	Moderate	Low	Serious
Beam et al., 2021 [30]	Low	Moderate	Low	Low	Low	Low	Moderate
Şahin-Bodur et al., 2021 [31]	Low	Low	Low	Low	Low	Low	Low
Tarçın et al., 2023 [5]	Low	Low	Low	Low	Low	Low	Low
Igudesman et al., 2023 [32]	Low	Moderate	Low	Moderate	Low	Low	Moderate
Yafei et al., 2023 [33]	Low	Low	Low	Low	Low	Moderate	Moderate
Ip et al., 2023 [34]	Low	Moderate	Serious	Low	Moderate	Low	Serious
Chou et al., 2023 [35]	Low	Moderate	Moderate	Low	Low	Low	Moderate
Hartlaub & Hill, 2025 [36]	Low	Moderate	Moderate	Low	Low	Low	Moderate
Oikonomou et al., 2025 [4]	Low	Low	Moderate	Low	Low	Low	Moderate
Figueiredo et al., 2026 [37]	Moderate	Moderate	Serious	Moderate	Moderate	Moderate	Serious
Ritz et al., 2026 [38]	Low	Moderate	Moderate	Moderate	Low	Low	Moderate
South et al., 2026 [2]	Low	Moderate	Moderate	Moderate	Moderate	Low	Moderate
	**Domains**: D1, bias in confounding; D2, bias in classification of intervention; D3, bias in selection into the study; D4, bias due to missing data; D5, bias in measurement of the outcome; D6, bias in selection of reported result. Legend for colour codes:						
Low risk	Low risk [except for concerns about uncontrolled confounding (only for D1)]				Moderate risk	Serious risk	Critical risk

## Data Availability

No new data were generated for this review; all data are in the published material of the studies that were referred to.

## Supplementary Materials

The following supporting information can be downloaded at: https://www.mdpi.com/article/10.3390/jcm15093518/s1, Table S1: Search strategy; Table S2: PRISMA 2020 Checklist; and Supplementary Figure S1. Risk of Bias assessment using the ROBINS-I V2 tool for all included records.

## Abbreviations

The following abbreviations are used in this manuscript:

BMI	Body mass index
DEB	Disordered eating behaviour
DEPS-R	Diabetes Eating Problem Survey–Revised
DKA	diabetic ketoacidosis
DSED	diabetes-specific eating disorder
DSM-5	Diagnostic and Statistical Manual of Mental Disorders
ED	eating disorder
EDE-Q	Eating Disorder Examination Questionnaire
HbA1c	glycated haemoglobin
ICD-10	International Classification of Diseases (World Health Organization)
IIO	intentional insulin omission
IR	insulin restriction
IRWC	insulin restriction for weight control
MDD	Major depressive disorder
PRISMA	Preferred Reporting Items for Systematic reviews and Meta-Analyses
SCFA	short-chain fatty acid
T1D	type 1 diabetes mellitus
