# Supplementary material for: Intentional Insulin Omission (Diabulimia) in Patients with Insulin-Dependent Diabetes: An Eating Disorder? A Systematic Review
Source: J Clin Med. 2026 May 4;15(9):3518. doi: 10.3390/jcm15093518 (PMC13163752; doi:10.3390/jcm15093518)
Supplement: Supplementary file 1 [file jcm-15-03518-s001.zip › jcm-4262857-supplementary.pdf]

Supplementary Table S1. Documents resulting from our database searches with eligibility and reasons for exclusion.

| diabulimia[ti] OR "insulin restriction"[ti] OR (skipping[ti] AND insulin[ti]) OR "insulin omission"[ti] PubMed 1.4.2026 → 60 results | | |
|---|---|---|
| 1 | Altabas V, Marinković Radošević J, Grubiješić N, A review on diabulimia: Exploring the intersection of disordered eating, eating disorders, insulin dose manipulation, and type 1 diabetes, Curr Diabetes Rev. 2026;22(5):e15733998347278. doi: 10.2174/0115733998347278250309180707. Online ahead of print 2025 Jul 14. | Review |
| **2** | **Oikonomou A, Christoforidis A, Kotanidou EP, Giannopoulou I, Paschalidou E, Tsinopoulou VR, Sotiriou G, Tsiroukidou K, Galli-Tsinopoulou A. Detecting disordered eating behaviors in Greek youth with type 1 diabetes mellitus by using the Diabetes Eating Problem Survey-Revised (DEPS-R): Associations with insulin restriction, glycemic control, and anthropometric parameters. Children (Basel). 2025;12(6):795. doi: 10.3390/children12060795.** | **Included** |
| 3 | Hartlaub JM, Hill EM. Disordered eating and intentional insulin restriction among young adults with type 1 diabetes: An examination of diabetes distress and appearance-related perceptions as correlates. Int J Eat Disord. 2025. doi: 10.1002/eat.24490. Online ahead of print Jun 21. | Included |
| 4 | Shchurenkov AV, Kazaryan PA, Rozhdestvenskaya VA, Poluboyarinova IV, Iuzbashian PG, Voronova EI, Fadeev VV, Romanov DV. Щуренков А.В., Казарян П.А., Рождественская В.А., Полубояринова И.В., Юзбашян П.Г., Воронова Е.И., Фадеев В.В., Романов Д.В. Клинический случай диабулимии при сахарном диабете 1 типа: психопатологические механизмы и психосоматические соотношения [A clinical case of diabulimia in type 1 diabetes mellitus: Psychopathological mechanisms and psychosomatic relationships]. Zh Nevrol Psikhiatr Im S S Korsakova. 2025;125(4):118-123 (Журнал неврологии и психиатрии им. С.С. Корсакова. 2025;125(4): 118-123). doi: 10.17116/jnevro2025125041118. Russian. | Case |
| 5 | Beam AB, Wiebe DJ. Subtypes of insulin restriction in diabetes management: A systematic review. Curr Diab Rep. 2025;25(1):20. doi: 10.1007/s11892-025-01577-3. | Review |
| 6 | Çelik ZM, Bayram HM. Unlocking the secrets: How well do nutrition and dietetics students understand diabulimia and health literacy? Nutr Health. 2025:2601060241310639. doi: 10.1177/02601060241310639. Online ahead of print Jan 19. | No pts |
| 7 | Wal A, Sahu PK, Wal P, Sahu K, Bhise MR, Lodhi DS. A comprehensive review of fear of eating behaviour in individuals with diabetes: Exploring therapeutic interventions for diabulimia. Cardiovasc Hematol Disord Drug Targets. 2024;24(4):218-227. doi: 10.2174/011871529X326042241031060350. | Review |
| 8 | Ng SJY, Goh ML. Reducing insulin omission errors among patients with diabetes mellitus in general surgical wards: a best practice implementation project. JBI Evid Implement. 2024;22(3):291-302. doi: 10.1097/XEB.0000000000000437. | No diabulimia |
| 9 | Rivolta B, Masserini B, Bernardi I, Camera A, Liboà F, Solerte SB, Cerabolini C, Cerutti N. Diabulimia and type 1 diabetes: An unknown and emerging problem. Endocr Metab Immune Disord Drug Targets. 2024. doi: 10.2174/0118715303314948240419060714. Online ahead of print Apr 30. | Case |
| 10 | Poos S, Faerovitch M, Pinto C, Jamalkhani N, Chaudhri F, Khan S, Lo DF, McGowan K, Martin A. The role of diabetes distress in Diabulimia. J Eat Disord. 2023;11(1):213. doi: 10.1186/s40337-023-00924-7. | Review |
| 11 | Çetinkaya Özdemir S, Semerci V, Sönmez Sarı E. Development of the diabulimia knowledge level scale. Arch Psychiatr Nurs. 2023;46:139-145. doi: 10.1016/j.apnu.2023.09.003. Epub 2023 Sep 18. | No pts |
| 12 | Kara A, Gerçek HG, Özkan Y, Çelikkol Sadıç Ç, Koca SB. Depression, anxiety, eating problems, and diabulimia risk in adolescents with type 1 diabetes: a case-control study. J Pediatr Endocrinol Metab. 2023;36(10):957-965. doi: 10.1515/jpem-2023-0330. Print 2023 Oct 26. | Unfocused |
| **13** | **Chou WC, Chou YY, Pan YW, Ou TY, Tsai MC. Correlates of disordered eating and insulin restriction behavior and its association with psychological health in Taiwanese youths with diabetes mellitus. J Eat Disord. 2023;11(1):158. doi: 10.1186/s40337-023-00888-8.** | **Included** |
| **14** | **Ip EJ, Doroudgar S, Salehi A, Salehi F, Najmi M. Diabulimia: A risky trend among adults with type 1 diabetes mellitus. Endocr Pract. 2023;29(11):849-854. doi: 10.1016/j.eprac.2023.08.001. Epub 2023 Aug 9.** | **Included** |
| 15 | Levek N, Faruge-Hadiga R, Pinhas-Hamiel O. Insulin omission for weight loss in a female adolescent treated with advanced hybrid closed-loop system: A word of caution. Diabetes Care. 2023;46(8):e143-e145. doi: 10.2337/dc23-0204. | Case |
| **16** | **Yafei S, Hummadi A, Badedi M, Darraj H, Khawaji A, Alzughbi T, Abutaleb R, Alhagawy AJ, Alnami A, Kudam B, Bahsan F, Kariri M, Adawi M, Daghriri M, Hassan R, Soeid M, Alzughbi N. Disordered eating behaviors and insulin restriction in Saudi adolescents and young adults with type 1 diabetes. Medicina (Kaunas). 2023;59(2):345. doi: 10.3390/medicina59020345.** | **Included** |
| 17 | Ferrey A, Ashworth G, Cabling M, Rundblad G, Ismail K. A thematic analysis of YouTube comments on a television documentary titled 'Diabulimia: The World's most dangerous eating disorder'. Diabet Med. 2023;40(5):e15025. doi: 10.1111/dme.15025. Epub 2022 Dec 19. | Unfocused |
| 18 | Bereda G. Case report: Diabetic ketoacidosis during pregnancy due to insulin omission. Open Access Emerg Med. 2022;14:615-618. doi: 10.2147/OAEM.S388941. eCollection Nov 15 2022. | Case |
| 19 | Goddard G, Oxlad M. Insulin restriction or omission in Type 1 Diabetes Mellitus: a meta-synthesis of individuals' experiences of diabulimia. Health Psychol Rev. 2023;17(2):227-246. doi: 10.1080/17437199.2021.2025133. Epub 2022 Jan 31. | Review |
| **20** | **Beam AB, Wiebe DJ, Berg CA. Insulin restriction, emotion dysregulation, and depressive symptoms in late adolescents with diabetes. J Pediatr Psychol. 2021;46(9):1110-1118. doi: 10.1093/jpepsy/jsab042.** | **Included** |
| 21 | Hall R, Keeble L, Sünram-Lea SI, To M. A review of risk factors associated with insulin omission for weight loss in type 1 diabetes. Clin Child Psychol Psychiatry. 2021;26(3):606-616. doi: 10.1177/13591045211026142. Epub 2021 Jun 13. | Review |
| 22 | Ferrero Franco R, Garcia de Lorenzo A, Gonzalez Castro A. Diabulimia: An updated perspective. Enferm Clin (Engl Ed). 2021;31(6):396-397. doi: 10.1016/j.enfcle.2021.02.003. Epub 2021 Jun 9. | Opinion |
| 23 | Ferrero Franco R, Garcia de Lorenzo A, Gonzalez Castro A. Diabulimia: An updated perspective. Enferm Clin (Engl Ed). 2021:S1130-8621(21)00053-X. doi: 10.1016/j.enfcli.2021.02.001. Online ahead of print Mar 6. English, Spanish. | Duplicate 22PM |
| **24** | **Coleman SE, Caswell N. Diabetes and eating disorders: an exploration of 'Diabulimia'. BMC Psychol. 2020;8(1):101. doi: 10.1186/s40359-020-00468-4.** | **Included** |
| 25 | Wisting L, Snoek F. Terminology matters: 'diabulimia' is insufficient to describe eating disorders in individuals with Type 1 diabetes. Diabet Med. 2020;37(6):1075-1076. doi: 10.1111/dme.14108. Epub 2019 Aug 23. | Opinion |
| 26 | Brookes G. Insulin restriction, medicalisation and the Internet. Commun Med. 2019;15(1):14-27. doi: 10.1558/cam.33067. | Unfocused |
| 27 | Torjesen I. Diabulimia: the world's most dangerous eating disorder. BMJ. 2019;364:l982. doi: 10.1136/bmj.l982. | Opinion |
| 28 | Chelvanayagam S, James J. What is diabulimia and what are the implications for practice? Br J Nurs. 2018;27(17):980-986. doi: 10.12968/bjon.2018.27.17.980. | Case |
| 29 | De Paoli T, Rogers PJ. Disordered eating and insulin restriction in type 1 diabetes: A systematic review and testable model. Eat Disord. 2018;26(4):343-360. doi: 10.1080/10640266.2017.1405651. Epub 2017 Nov 28. | Review |
| 30 | Candler T, Murphy R, Pigott A, Gregory JW. Fifteen-minute consultation: Diabulimia and disordered eating in childhood diabetes. Arch Dis Child Educ Pract Ed. 2018;103(3):118-123. doi: 10.1136/archdischild-2017-312689. Epub 2017 Oct 27. | Review |
| 31 | Falcão MA, Francisco R. Diabetes, eating disorders and body image in young adults: an exploratory study about "diabulimia". Eat Weight Disord. 2017;22(4):675-682. doi: 10.1007/s40519-017-0406-9. Epub 2017 Jun 8. | Unfocused |
| 32 | Kınık MF, Gönüllü FV, Vatansever Z, Karakaya I. Diabulimia, a Type I diabetes mellitus-specific eating disorder. Turk Pediatri Ars. 2017;52(1):46-49. doi: 10.5152/TurkPediatriArs.2017.2366. eCollection Mar 1 2017. | Case |
| 33 | Wisting L, Reas DL, Bang L, Skrivarhaug T, Dahl-Jørgensen K, Rø Ø. Eating patterns in adolescents with type 1 diabetes: Associations with metabolic control, insulin omission, and eating disorder pathology. Appetite. 2017;114:226-231. doi: 10.1016/j.appet.2017.03.035. Epub 2017 Mar 27. | Included |
| 34 | Weiner S. The truth about diabulimia. Diabetes Self Manag. 2017;34(2):32-35. | Opinion |
| 35 | Hastings A, McNamara N, Allan J, Marriott M. The importance of social identities in the management of and recovery from 'Diabulimia': A qualitative exploration. Addict Behav Rep. 2016;4:78-86. doi: 10.1016/j.abrep.2016.10.003. eCollection Oct 15 2016. | Unfocused |
| **36** | **Bächle C, Stahl-Pehe A, Rosenbauer J. Disordered eating and insulin restriction in youths receiving intensified insulin treatment: Results from a nationwide population-based study. Int J Eat Disord. 2016;49(2):191-6. doi: 10.1002/eat.22463. Epub 2015 Sep 23.** | **Included** |
| **37** | **Merwin RM, Dmitrieva NO, Honeycutt LK, Moskovich AA, Lane JD, Zucker NL, Surwit RS, Feinglos M, Kuo J. Momentary predictors of insulin restriction among adults with type 1 diabetes and eating disorder symptomatology. Diabetes Care. 2015;38(11):2025-32. doi: 10.2337/dc15-0753. Epub 2015 Sep 17.** | **Included** |
| 38 | Davidson J. Diabulimia: how eating disorders can affect adolescents with diabetes. Nurs Stand. 2014;29(2):44-9. doi: 10.7748/ns.29.2.44.e7877. | Opinion |
| **39** | **Pinhas-Hamiel O, Hamiel U, Greenfield Y, Boyko V, Graph-Barel C, Rachmiel M, Lerner-Geva L, Reichman B. Detecting intentional insulin omission for weight loss in girls with type 1 diabetes mellitus. Int J Eat Disord. 2013;46(8):819-25. doi: 10.1002/eat.22138. Epub 2013 May 15.** | **Included** |
| 40 | Neithercott T. Body wars. Skipping meals, purging food, avoiding insulin--the scary world of eating disorders and diabetes. Diabetes Forecast. 2013;66(3):48-53. | Opinion |
| 41 | Murdoff L. Insulin omission for weight loss. The dangers of diabulimia. Adv NPs PAs. 2011;2(5):35-7, 50. | Opinion |

| | | |
|---|---|---|
| 42 | Haagen BF. Insulin omission. A troubling trend among adolescent girls. J Psychosoc Nurs Ment Health Serv. 2011;49(2):6-7. doi: 10.3928/02793695-20110116-01. | Opinion |
| **43** | **Goebel-Fabbri AE, Anderson BJ, Fikkan J, Franko DL, Pearson K, Weinger K. Improvement and emergence of insulin restriction in women with type 1 diabetes. Diabetes Care. 2011;34(3):545-50. doi: 10.2337/dc10-1547. Epub 2011 Jan 25.** | **Included** |
| 44 | Hasken J, Kresl L, Nydegger T, Temme M. Diabulimia and the role of school health personnel. J Sch Health. 2010;80(10):465-9; quiz 514-6. doi: 10.1111/j.1746-1561.2010.00529.x. | Review |
| 45 | Uthoff H, Lehmann R, Sprenger M, Wiesli P. Skipping meals or carbohydrate-free meals in order to determine Basal insulin requirements in subjects with type 1 diabetes mellitus? Exp Clin Endocrinol Diabetes. 2010;118(5):325-7. doi: 10.1055/s-0029-1241199. Epub 2010 Jan 12. | Unrelated |
| 46 | Ruth-Sahd LA, Schneider M, Haagen B. Diabulimia: what it is and how to recognize it in critical care. Dimens Crit Care Nurs. 2009;28(4):147-53; quiz 154-5. doi: 10.1097/DCC.0b013e3181a473fe. | Case |
| 47 | Alexander KE, Ventura EE, Spruijt-Metz D, Weigensberg MJ, Goran MI, Davis JN. Association of breakfast skipping with visceral fat and insulin indices in overweight Latino youth. Obesity (Silver Spring). 2009;17(8):1528-33. doi: 10.1038/oby.2009.127. Epub 2009 May 7. | Unrelated |
| 48 | Baginsky P. A battle to overcome "diabulimia". Am Fam Physician. 2009;79(4):263; discussion 263. | Case |
| 49 | Øverby NC, Margeirsdottir HD, Brunborg C, Dahl-Jørgensen K, Andersen LF; Norwegian Study Group for Childhood Diabetes. Sweets, snacking habits, and skipping meals in children and adolescents on intensive insulin treatment. Pediatr Diabetes. 2008;9(4 Pt 2):393-400. doi: 10.1111/j.1399-5448.2008.00381.x. | Unrelated |
| **50** | **Takii M, Uchigata Y, Tokunaga S, Amemiya N, Kinukawa N, Nozaki T, Iwamoto Y, Kubo C. The duration of severe insulin omission is the factor most closely associated with the microvascular complications of Type 1 diabetic females with clinical eating disorders. Int J Eat Disord. 2008;41(3):259-64. doi: 10.1002/eat.20498.** | **Included** |
| **51** | **Goebel-Fabbri AE, Fikkan J, Franko DL, Pearson K, Anderson BJ, Weinger K. Insulin restriction and associated morbidity and mortality in women with type 1 diabetes. Diabetes Care. 2008;31(3):415-9. doi: 10.2337/dc07-2026. Epub 2007 Dec 10.** | **Included** |
| 52 | Yan L. 'Diabulimia' a growing problem among diabetic girls. Nephrol News Issues. 2007;21(11):36, 38. | Case |
| 53 | Editorial. Skipping the syringe: options for insulin. Johns Hopkins Med Lett Health After 50. 2006;17(12):6. | Opinion |
| 54 | Mimasaka S, Funayama M, Azumi J, Morita M. Child death related to insulin omission by mother. J Clin Forensic Med. 1998;5(2):72-6. doi: 10.1016/s1353-1131(98)90057-8. | Case |
| 55 | Affenito SG, Adams CH. Are eating disorders more prevalent in females with type 1 diabetes mellitus when the impact of insulin omission is considered? Nutr Rev. 2001;59(6):179-82. doi: 10.1111/j.1753-4887.2001.tb07010.x. | Review |
| **56** | **Takii M, Komaki G, Uchigata Y, Maeda M, Omori Y, Kubo C. Differences between bulimia nervosa and binge-eating disorder in females with type 1 diabetes: the important role of insulin omission. J Psychosom Res. 1999;47(3):221-31. doi: 10.1016/s0022-3999(99)00031-8.** | **Included** |
| **57** | **Khan Y, Montgomery AMJ. Eating attitudes in young females with diabetes: insulin omission identifies a vulnerable subgroup. Br J Med Psychol. 1996;69(4):343-53. doi: 10.1111/j.2044-8341.1996.tb01877.x.** | **Included** |
| **58** | **Polonsky WH, Anderson BJ, Lohrer PA, Aponte JE, Jacobson AM, Cole CF. Insulin omission in women with IDDM. Diabetes Care. 1994;17(10):1178-85. doi: 10.2337/diacare.17.10.1178.** | **Included** |
| **59** | **Ritz PJ, Aguayo GA, Cosson E, Canha D, Renard E, Merwin RM, Amouyal C, Arnault G, Bilariki K, Borot S, Chevalier N, Lemoine A, Franc S, Frémy B, Gouet D, Julla JB, Marchand L, Pinto S, Rigalleau V, Sonnet E, Tatulashvili S, Tauveron I, Riveline JP, Hanaire H, Fagherazzi G. Association of eating disorders and/or insulin omission with impaired glycaemic control in persons living with type 1 diabetes: cross-sectional analysis of the French SFDT1 study. BMJ Open. 2026;16(3):e104542. doi: 10.1136/bmjopen-2025-104542.** | **Included** |
| **60** | **South CA, Brazeau AS, Talbo MK, Bandini A, Kichler JC, Iceta S. Factors associated with intentional insulin omission to lose weight in people living with type 1 diabetes: A BETTER registry analysis. Can J Diabetes. 2026:S1499-2671(26)00061-4. doi: 10.1016/j.jcjd.2026.03.004. Epub ahead of print 2026 Mar 25.** | **Included** |
| TITLE-ABS-KEY ( diabulimia OR insulin restriction OR skipping insulin OR insulin omission ) Scopus 1.4.2026 → 53 documents | | |
| 61 | Altabas V, Marinković Radošević J, Grubiješić N, A review on diabulimia: Exploring the intersection of disordered eating, eating disorders, insulin dose manipulation, and type 1 diabetes, Curr Diabetes Rev. 2026;22(5):e15733998347278. doi: 10.2174/0115733998347278250309180707. Online ahead of print 2025 Jul 14. | Dupl 1PM |
| 62 | Lasher AT, Heckman B, Sarker P, Liu K, Sun LY. Uncoupling Insulin Sensitivity From Longevity: A Sex-Dependent Effect of Hepatic Glucagon Signaling. Aging Cell. 2026;25(1):e70349. doi: 10.1111/acel.70349. | Animal |
| **63** | **Figueiredo JdCM, João Pavin E, Trevisan TL, Vilela da Mota Silveira MS. Disordered eating behaviors in persons with type 1 diabetes: frequency, risk factors, depressive and anxiety symptoms, and clinical outcomes. Eat Disord. 2026:1-16. doi: 10.1080/10640266.2026.2618759. Epub ahead of print 2026 Jan 29.** | **Included** |
| 64 | Beam AB, Wiebe DJ. Subtypes of insulin restriction in diabetes management: A systematic review. Curr Diab Rep. 2025;25(1):20. doi: 10.1007/s11892-025-01577-3. | Dupl 5PM |
| 65 | Marques SJS, Garces TS, Mattos SM, Damasceno LLV, Araújo AL, Forte RSC, Freire KA, Cestari VRF, Freitas CHA, Moreira TMM. Disordered eating behaviors in people with type 1 diabetes mellitus: a scoping review. Rev Esc Enferm USP. 2025 Dec 8;59:e20250177. doi: 10.1590/1980-220X-REEUSP-2025-0177en. | Review |
| 66 | Afeef S, Zakrzewski-Fruer JK, Thackray AE, Barrett LA, Tolfrey K. Impact of breakfast consumption timing v. breakfast omission on post-lunch glycaemia and insulinaemia in adolescent girls: a randomised crossover trial. Br J Nutr. 2025;133(5):611-622. doi: 10.1017/S0007114525000248. Epub 2025 Feb 14. | Unrelated |
| 67 | Dobbins RA, Salmon AL. Psychological impact of type 1 diabetes: A focus on disordered eating behavior in female adolescents. Diabetes Spectr. 2025;38(5):543-549. doi: 10.2337/ds25-0001. | Review |
| 68 | Partridge H, Figueiredo C, Chapman S. The complex interplay of type 1 diabetes and eating disorders. In: Robinson P, Wade T, Herpertz-Dahlmann B, Fernandez-Aranda F, Treasure J, Wonderlich S (eds) Eating Disorders. Cham, CH: Springer, 2024, pp. 593-606. doi: 10.1007/978-3-030-97416-9_36-1. | Review |
| 69 | Clayton DJ, Varley I, Papageorgiou M. Intermittent fasting and bone health: a bone of contention? Br J Nutr. 2023 Nov 14;130(9):1487-1499. doi: 10.1017/S0007114523000545. Epub 2023 Mar 6. | Unrelated |
| 70 | Ip EJ, Doroudgar S, Salehi A, Salehi F, Najmi M. Diabulimia: A risky trend among adults with type 1 diabetes mellitus. Endocr Pract. 2023;29(11):849-854. doi: 10.1016/j.eprac.2023.08.001. Epub 2023 Aug 9. | Dupl 14PM |
| 71 | Jaensch L, Goddard G, Oxlad M, Franke E. Health professionals' experiences supporting people with type 1 diabetes mellitus who deliberately restrict and/or omit insulin for weight, shape, and/or appearance: A meta-synthesis. Can J Diabetes. 2023;47(6):532-542. doi: 10.1016/j.jcjd.2023.03.003. Epub 2023 Mar 28. | Review |
| 72 | Goddard G, Oxlad M, Turnbull D. The misuse of insulin by males with Type 1 Diabetes Mellitus for weight and/or shape control: a systematic scoping review. J Diabetes Metab Disord. 2022;22(1):13-34. doi: 10.1007/s40200-022-01151-8. | Review |
| 73 | Goddard G, Oxlad M. Insulin restriction or omission in Type 1 Diabetes Mellitus: a meta-synthesis of individuals' experiences of diabulimia. Health Psychol Rev. 2023;17(2):227-246. doi: 10.1080/17437199.2021.2025133. Epub 2022 Jan 31. | Dupl 19PM |
| 74 | Goddard G, Oxlad M. Caring for individuals with Type 1 Diabetes Mellitus who restrict and omit insulin for weight control: Evidence-based guidance for healthcare professionals. Diabetes Res Clin Pract. 2022;185:109783. doi: 10.1016/j.diabres.2022.109783. Epub 2022 Feb 17. | Review |
| 75 | Camelo Rebouças KS, Lopes FKdM, Teixeira Martins MYP, Jataí Silva R, Martins Amorim AK, Luna Queiroz L, Tavares Ramos LT, Costa Maia CS, Cavalcante Lopes S, Montenegro Júnior RM, Vasconcelos Albuquerque N. Omissão de refeições, estado nutricional e perfil metabólico de pacientes com diabetes mellitus tipo 2 [Omission of meals, nutritional status and metabolic profile of patients with type 2 diabetes mellitus]. Nutrición Clínica y Dietética Hospitalaria. 2022;42(3):152-9. | Unrelated |
| 76 | Beam AB, Wiebe DJ, Berg CA. Insulin restriction, emotion dysregulation, and depressive symptoms in late adolescents with diabetes. J Pediatr Psychol. 2021;46(9):1110-1118. doi: 10.1093/jpepsy/jsab042. | Dupl 20PM |
| 77 | Francisco R. Type 1 diabetes and disordered eating behavior. Manzato E, Cuzzolaro M, Donini LM (eds.), Hidden and Lesser-known Disordered Eating Behaviors in Medical and Psychiatric Conditions. Cham, CH: Springer, 2021; Ch. 25, pp. 253-259. doi:10.1007/978-3-030-81174-7_25 | Review |
| 78 | Lindgren O, Ahrén B. Consequences on islet and incretin hormone responses to dinner by omission of lunch in healthy men. Endocrinol Diabetes Metab. 2020 Apr 28;3(3):e00141. doi: 10.1002/edm2.141. | Unrelated |
| 79 | Ayalew MB, Dieberg G, Quirk F, Spark MJ. Potentially inappropriate prescribing for adults with diabetes mellitus: a scoping review protocol. JBI Evid Synth. 2020 Jul;18(7):1557-1565. doi: 10.11124/JBISRIR-D-19-00136. | Review |
| 80 | Stekovic S, Hofer SJ, Tripolt N, Aon MA, Royer P, Pein L, Stadler JT, Pendl T, Prietl B, Url J, Schroeder S, Tadic J, Eisenberg T, Magnes C, Stumpe M, Zuegner E, Bordag N, Riedl R, Schmidt A, Kolesnik E, Verheyen N, Springer A, Madl T, Sinner F, de Cabo R, Kroemer G, Obermayer-Pietsch B, Dengjel J, | Unrelated |

| | | |
|---|---|---|
| | Sourij H, Pieber TR, Madeo F. Alternate day fasting improves physiological and molecular markers of aging in healthy, non-obese humans. Cell Metab. 2019;30(3):462-476.e6. doi: 10.1016/j.cmet.2019.07.016. Epub 2019 Aug 27. Erratum in: Cell Metab. 2020;31(4):878-881. doi: 10.1016/j.cmet.2020.02.011. | |
| 81 | Hernández Rodríguez J, Ledón Llanes L. Comportamiento clínico y enfoque terapéutico de los trastornos alimentarios en personas con diabetes mellitus tipo 1 [Clinical behavior and therapeutic approach of eating disorders in people with type 1 diabetes mellitus]. Revista Cubana de Medicina General Integral, 2020;36(2):e1280, 1-15. | Review |
| 82 | Stein D, Keller S, Ifergan IS, Shilton T, Toledano A, Pelleg MT, Witztum E. Extreme risk-taking behaviors in patients with eating disorders. Front Psychiatry. 2020;11:89. doi: 10.3389/fpsyt.2020.00089. Epub Feb 28 2020. | Case |
| 83 | Phillips E. Co-occurring eating disorders and type 1 diabetes mellitus. In Evans YN, Dixon Docter A (Eds.) Adolescent Nutrition. Assuring the Needs of Emerging Adults. Cham CH: Springer, 2020; pp. 589-616. doi: 10.1007/978-3-030-45103-5_19 | Review |
| 84 | Haslacher H, Fallmann H, Waldhäusl C, Hartmann E, Wagner OF, Waldhäusl W. Type 2 diabetes care: Improvement by standardization at a diabetes rehabilitation clinic. An observational report. PLoS One. 2019;14(12):e0226132. doi: 10.1371/journal.pone.0226132. | Unrelated |
| **85** | **Luyckx K, Verschueren M, Palmeroni N, Goethals ER, Weets I, Claes L. Disturbed eating behaviors in adolescents and emerging adults with type 1 diabetes: A one-year prospective study. Diabetes Care. 2019;42(9):1637-1644. doi: 10.2337/dc19-0445. Epub 2019 Jun 19.** | **Included** |
| 86 | Ogata H, Hatamoto Y, Goto Y, Tajiri E, Yoshimura E, Kiyono K, Uehara Y, Kawanaka K, Omi N, Tanaka H. Association between breakfast skipping and postprandial hyperglycaemia after lunch in healthy young individuals. Br J Nutr. 2019 Aug 28;122(4):431-440. doi: 10.1017/S0007114519001235. | Unrelated |
| 87 | Staite E, Zaremba N, Macdonald P, Allan J, Treasure J, Ismail K, Stadler M. 'Diabulimia' through the lens of social media: a qualitative review and analysis of online blogs by people with Type 1 diabetes mellitus and eating disorders. Diabet Med. 2018;35(10):1329-1336. doi: 10.1111/dme.13700. Epub 2018 Jun 20. | No pts |
| 88 | Adams WK, D'souza AM, Sussman JL, Kaur S, Kieffer TJ, Winstanley CA. Enhanced amphetamine-induced motor impulsivity and mild attentional impairment in the leptin-deficient rat model of obesity. Physiol Behav. 2018;192:134-144. doi: 10.1016/j.physbeh.2018.03.027. Epub 2018 Mar 27. | Animal |
| 89 | De Paoli T, Rogers PJ. Disordered eating and insulin restriction in type 1 diabetes: A systematic review and testable model. Eat Disord. 2018;26(4):343-360. doi: 10.1080/10640266.2017.1405651. Epub 2017 Nov 28. | Dupl 29PM |
| 90 | Jakubowicz D, Wainstein J, Landau Z, Raz I, Ahren B, Chapnik N, Ganz T, Menaged M, Barnea M, Bar-Dayan Y, Froy O. Influences of breakfast on clock gene expression and postprandial glycemia in healthy individuals and individuals with diabetes: A randomized clinical trial. Diabetes Care. 2017;40(11):1573-1579. doi: 10.2337/dc16-2753. Epub 2017 Aug 22. | Unrelated |
| 91 | Wisting L, Reas DL, Bang L, Skrivarhaug T, Dahl-Jørgensen K, Rø Ø. Eating patterns in adolescents with type 1 diabetes: Associations with metabolic control, insulin omission, and eating disorder pathology. Appetite. 2017;114:226-231. doi: 10.1016/j.appet.2017.03.035. Epub 2017 Mar 27. | Dupl 33PM |
| 92 | Clayton DJ, Stensel DJ, James LJ. Effect of breakfast omission on subjective appetite, metabolism, acylated ghrelin and GLP-17-36 during rest and exercise. Nutrition. 2016;32(2):179-85. doi: 10.1016/j.nut.2015.06.013. Epub 2015 Jul 22. | Unrelated |
| 93 | Chowdhury EA, Richardson JD, Tsintzas K, Thompson D, Betts JA. Effect of extended morning fasting upon ad libitum lunch intake and associated metabolic and hormonal responses in obese adults. Int J Obes (Lond). 2016;40(2):305-11. doi: 10.1038/ijo.2015.154. Epub 2015 Aug 17. | Unrelated |
| 94 | Clayton DJ, Barutcu A, Machin C, Stensel DJ, James LJ. Effect of Breakfast Omission on Energy Intake and Evening Exercise Performance. Med Sci Sports Exerc. 2015;47(12):2645-52. doi: 10.1249/MSS.0000000000000702. | Unrelated |
| 95 | Chowdhury EA, Richardson JD, Tsintzas K, Thompson D, Betts JA. Carbohydrate-rich breakfast attenuates glycaemic, insulinaemic and ghrelin response to ad libitum lunch relative to morning fasting in lean adults. Br J Nutr. 2015;114(1):98-107. doi: 10.1017/S0007114515001506. Epub 2015 May 25. | Unrelated |
| 96 | Elleri D, Maltoni G, Allen JM, Nodale M, Kumareswaran K, Leelarathna L, Thabit H, Caldwell K, Wilinska ME, Calhoun P, Kollman C, Dunger DB, Hovorka R. Safety of closed-loop therapy during reduction or omission of meal boluses in adolescents with type 1 diabetes: a randomized clinical trial. Diabetes Obes Metab. 2014;16(11):1174-8. doi: 10.1111/dom.12324. Epub 2014 Jul 6. | Unrelated |
| 97 | Hanlan ME, Griffith J, Patel N, Jaser SS. Eating disorders and disordered eating in type 1 diabetes: Prevalence, screening, and treatment options. Curr Diab Rep. 2013;13(6):909-916. doi: 10.1007/s11892-013-0418-4. Epub ahead of print 2013 Sep 12. | Review |
| **98** | **Wisting L, Frøisland DH, Skrivarhaug T, Dahl-Jørgensen K, Rø O. Disturbed eating behavior and omission of insulin in adolescents receiving intensified insulin treatment: a nationwide population-based study. Diabetes Care. 2013;36(11):3382-7. doi: 10.2337/dc13-0431. Epub 2013 Aug 20.** | **Included** |
| 99 | Pinhas-Hamiel O, Levy-Shraga Y. Eating disorders in adolescents with type 2 and type 1 diabetes. Curr Diab Rep. 2013;13(2):289-97. doi: 10.1007/s11892-012-0355-7. | Review |
| 100 | Besser REJ, Jones AG, McDonald TJ, Shields BM, Knight BA, Hattersley AT. The impact of insulin administration during the mixed meal tolerance test. Diabet Med. 2012;29(10):1279-84. doi: 10.1111/j.1464-5491.2012.03649.x. | Unfocused |
| 101 | King AB, Clark D, Wolfe GS. Contribution of the dawn phenomenon to the fasting and postbreakfast hyperglycemia in type 1 diabetes treated with once-nightly insulin glargine. Endocr Pract. 2012;18(4):558-62. doi: 10.4158/EP12042.OR. | Unrelated |
| **102** | **Peyrot M, Rubin RR, Kruger DF, Travis LB. Correlates of insulin injection omission. Diabetes Care. 2010;33(2):240-5. doi: 10.2337/dc09-1348.** | **Included** |
| 103 | Alexander KE, Ventura EE, Spruijt-Metz D, Weigensberg MJ, Goran MI, Davis JN. Association of breakfast skipping with visceral fat and insulin indices in overweight Latino youth. Obesity (Silver Spring). 2009;17(8):1528-33. doi: 10.1038/oby.2009.127. Epub 2009 May 7. | Dupl 47PM |
| **104** | **Adisa R, Alutundu MB, Fakeye TO. Factors contributing to nonadherence to oral hypoglycemic medications among ambulatory type 2 diabetes patients in Southwestern Nigeria. Pharm Pract (Granada). 2009;7(3):163-9. doi: 10.4321/s1886-36552009000300006. Epub 2009 Mar 15.** | **Included** |
| 105 | Johnstone HC, McNally RJQ, Cheetham TD. The impact of fasting and treatment omission on susceptibility to hypoglycaemia in children and adolescents with GH and cortisol insufficiency. Clin Endocrinol (Oxf). 2008;69(3):436-42. doi: 10.1111/j.1365-2265.2008.03210.x. Epub 2008 Feb 1. | No diab |
| **106** | **Ackard DM, Vik N, Neumark-Sztainer D, Schmitz KH, Hannan P, Jacobs DR Jr. Disordered eating and body dissatisfaction in adolescents with type 1 diabetes and a population-based comparison sample: comparative prevalence and clinical implications. Pediatr Diabetes. 2008;9(4 Pt 1):312-9. doi: 10.1111/j.1399-5448.2008.00392.x. Epub 2008 May 7.** | **Included** |
| 107 | Shi Y, Guo M, Yan J, Sun W, Zhang X, Geng L, Xu L, Chen Z. Analysis of clinical characteristics in large-scale Chinese women with polycystic ovary syndrome. Neuro Endocrinol Lett. 2007;28(6):807-10. | Unrelated |
| 108 | Alavi A-A, Amirhakimi E, Karami B. The prevalence of dental caries in 5 - 18-year-old insulin-dependent diabetics of Fars Province, southern Iran. Arch Iran Med. 2006 Jul;9(3):254-60. | Unrelated |
| 109 | Yang HX, Gao XL, Dong Y, Shi CY. Analysis of oral glucose tolerance test in pregnant women with abnormal glucose metabolism. Chin Med J (Engl). 2005;118(12):995-9. | Unrelated |
| 110 | Nissim R, Rodin G, Daneman D, Rydall A, Colton P, Maharaj S, Jones J. (רינת ניסים, גארי רודין, דניס דנמן, אן ריידל, פטרישיה קולטון, שרי מהרז 'וג'ניפר ג'ונס) הפרעות אכילה בקרב מתבגרות חולות סוכרת מסוג 1 (hafraot achila bakrav mitbagrot holot sukaret mesug 1) [Eating disturbances in adolescent girls with type 1 diabetes mellitus]. Harefuah (הרפואה). 141;2002(10):929, 902-7. Hebrew. | Review |
| 111 | Crow SJ, Keel PK, Kendall D. Eating disorders and insulin-dependent diabetes mellitus. Psychosomatics. 1998;39(3):233-43. doi: 10.1016/S0033-3182(98)71340-4. | Review |
| 112 | Khan Y, Montgomery AMJ. Eating attitudes in young females with diabetes: insulin omission identifies a vulnerable subgroup. Br J Med Psychol. 1996;69(4):343-53. doi: 10.1111/j.2044-8341.1996.tb01877.x. | Dupl 57PM |
| 113 | Castillo M, Campillo JE, Martinez Valdivia M, Osorio C. Effect of phosphate omission on the glucose-induced insulin release in vitro in fed and fasted rats. Acta Diabetol Lat. 1982;19(3):281-3. doi: 10.1007/BF02624688. | Animal |
| TI diabulimia OR TI insulin restriction OR TI skipping insulin OR TI insulin omission 1.4.2026 APA PsycINFO/PsycARTICLES → 37 Documents | | |
| 114 | Hartlaub JM, Hill EM. Disordered Eating and Intentional Insulin Restriction Among Young Adults With Type 1 Diabetes: An Examination of Diabetes Distress and Appearance-Related Perceptions as Correlates. Int J Eat Disord. 2025. doi: 10.1002/eat.24490. Epub ahead of print Jun 21. | Dupl 3PM |
| 115 | Zhao X, Lu J, Zhang J, Liu C, Wang H, Wang Y, Du Q. Sleep restriction promotes brain oxidative stress and inflammation, and aggravates cognitive impairment in insulin-resistant mice. Psychoneuroendocrinology. 2024;166:107065. doi: 10.1016/j.psyneuen.2024.107065. Epub 2024 Apr 29. | Animal |
| 116 | Kennon F, Robinson G. Parenting a child with 'diabulimia': A systemic interpretative phenomenological analysis. J Fam Ther. 2024;46(3):245-263 | Case |
| 117 | Poos S, Faerovitch M, Pinto C, Jamalkhani N, Chaudhri F, Khan S, Lo DF, McGowan K, Martin A. The role of diabetes distress in Diabulimia. J Eat Disord. 2023;11(1):213. doi: 10.1186/s40337-023-00924-7. | Dupl 10PM |
| 118 | Çetinkaya Özdemir S, Semerci V, Sönmez Sarı E. Development of the diabulimia knowledge level scale. Arch Psychiatr Nurs. 2023;46:139-145. doi: 10.1016/j.apnu.2023.09.003. Epub 2023 Sep 18. | Dupl 11PM |
| 119 | Chou WC, Chou YY, Pan YW, Ou TY, Tsai MC. Correlates of disordered eating and insulin restriction behavior and its association with psychological health in Taiwanese youths with diabetes mellitus. J Eat Disord. 2023;11(1):158. doi: 10.1186/s40337-023-00888-8. | Dupl 13PM |

| | | |
|---|---|---|
| 120 | Qi W, Gundogan F, Gilligan J, Monte S. Dietary soy prevents fetal demise, intrauterine growth restriction, craniofacial dysmorphic features, and impairments in placentation linked to gestational alcohol exposure: Pivotal role of insulin and insulin-like growth factor signaling networks. Alcohol. 2023 Aug;110:65-81. doi: 10.1016/j.alcohol.2023.01.006. Epub 2023 Mar 9. | Animal |
| 121 | Goddard G, Oxlad M. Insulin restriction or omission in Type 1 Diabetes Mellitus: a meta-synthesis of individuals' experiences of diabulimia. Health Psychol Rev. 2023;17(2):227-246. doi: 10.1080/17437199.2021.2025133. Epub 2022 Jan 31. | Dupl 19PM 8Sc |
| 122 | Ahsan, Zafina. Diabulimia: A systematic review of treatment. Dissertation Abstracts International: Section B: The Sciences and Engineering, 2023 | Review |
| 123 | Beam AB, Wiebe DJ, Berg CA. Insulin Restriction, Emotion Dysregulation, and Depressive Symptoms in Late Adolescents with Diabetes. J Pediatr Psychol. 2021;46(9):1110-1118. doi: 10.1093/jpepsy/jsab042. | Dupl 20PM 11Sc |
| 124 | Hall R, Keeble L, Sünram-Lea SI, To M. A review of risk factors associated with insulin omission for weight loss in type 1 diabetes. Clin Child Psychol Psychiatry. 2021;26(3):606-616. doi: 10.1177/13591045211026142. Epub 2021 Jun 13. | Dupl 21PM |
| 125 | Coleman SE, Caswell N. Diabetes and eating disorders: an exploration of 'Diabulimia'. BMC Psychol. 2020;8(1):101. doi: 10.1186/s40359-020-00468-4. | Dupl 24PM |
| 126 | Oki K, Arias EB, Kanzaki M, Cartee GD. Effects of Acute Exercise Combined With Calorie Restriction Initiated Late-in-Life on Insulin Signaling, Lipids, and Glucose Uptake in Skeletal Muscle From Old Rats. J Gerontol A Biol Sci Med Sci. 2020;75(2):207-217. doi: 10.1093/gerona/gly222. | Animal |
| 127 | De Paoli T, Rogers PJ. Disordered eating and insulin restriction in type 1 diabetes: A systematic review and testable model. Eat Disord. 2018;26(4):343-360. doi: 10.1080/10640266.2017.1405651. Epub 2017 Nov 28. | Dupl 29PM 24Sc |
| 128 | Falcão MA, Francisco R. Diabetes, eating disorders and body image in young adults: an exploratory study about "diabulimia". Eat Weight Disord. 2017;22(4):675-682. doi: 10.1007/s40519-017-0406-9. Epub 2017 Jun 8. | Dupl 31PM |
| 129 | Wisting L, Reas DL, Bang L, Skrivarhaug T, Dahl-Jørgensen K, Rø Ø. Eating patterns in adolescents with type 1 diabetes: Associations with metabolic control, insulin omission, and eating disorder pathology. Appetite. 2017;114:226-231. doi: 10.1016/j.appet.2017.03.035. Epub 2017 Mar 27. | Dupl 33PM 26Sc |
| 130 | Hastings A, McNamara N, Allan J, Marriott M. The importance of social identities in the management of and recovery from 'Diabulimia': A qualitative exploration. Addict Behav Rep. 2016;4:78-86. doi: 10.1016/j.abrep.2016.10.003. eCollection Oct 15 2016. | Dupl 35PM |
| 131 | Bächle C, Stahl-Pehe A, Rosenbauer J. Disordered eating and insulin restriction in youths receiving intensified insulin treatment: Results from a nationwide population-based study. Int J Eat Disord. 2016;49(2):191-6. doi: 10.1002/eat.22463. Epub 2015 Sep 23. | Dupl 36PM |
| 132 | Camps SGJA, Verhoef SPM, Westerterp KR. Physical activity and weight loss are independent predictors of improved insulin sensitivity following energy restriction. Obesity (Silver Spring). 2016;24(2):291-6. doi: 10.1002/oby.21325. Epub 2016 Jan 6. | Unfocused |
| 133 | Leproult R, Deliens G, Gilson M, Peigneux P. Beneficial impact of sleep extension on fasting insulin sensitivity in adults with habitual sleep restriction. Sleep. 2015 May 1;38(5):707-15. doi: 10.5665/sleep.4660. | Unrelated |
| 134 | Schwimmer, Katherine J. Insulin omission in adolescents with type I diabetes: A model group intervention. Dissertation Abstracts International: Section B: The Sciences and Engineering, 2015. The California School of Professional Psychology, San Francisco Campus, Alliant International University, San Diego. | Unfocused |
| 135 | Pinhas-Hamiel O, Hamiel U, Greenfield Y, Boyko V, Graph-Barel C, Rachmiel M, Lerner-Geva L, Reichman B. Detecting intentional insulin omission for weight loss in girls with type 1 diabetes mellitus. Int J Eat Disord. 2013;46(8):819-25. doi: 10.1002/eat.22138. Epub 2013 May 15. | Dupl 39PM |
| 136 | Klingenberg L, Chaput JP, Holmbäck U, Visby T, Jennum P, Nikolic M, Astrup A, Sjödin A. Acute Sleep Restriction Reduces Insulin Sensitivity in Adolescent Boys. Sleep. 2013;36(7):1085-1090. doi: 10.5665/sleep.2816. | Unrelated |
| 137 | Wang KCW, Zhang L, McMillen IC, Botting KJ, Duffield JA, Zhang S, Suter CM, Brooks DA, Morrison JL. Fetal growth restriction and the programming of heart growth and cardiac insulin-like growth factor 2 expression in the lamb. J Physiol. 2011;589(Pt 19):4709-22. doi: 10.1113/jphysiol.2011.211185. Epub 2011 Aug 1. | Animal |
| 138 | Haagen BF. Insulin omission. A troubling trend among adolescent girls. J Psychosoc Nurs Ment Health Serv. 2011;49(2):6-7. doi: 10.3928/02793695-20110116-01. | Dupl 42PM |
| 139 | Kauffman AL, Ashraf JM, Corces-Zimmerman MR, Landis JN, Murphy CT. Insulin signaling and dietary restriction differentially influence the decline of learning and memory with age. PLoS Biol. 2010;8(5):e1000372. doi: 10.1371/journal.pbio.1000372. | Animal |
| 140 | Muhlhausler BS, Duffield JA, Ozanne SE, Pilgrim C, Turner N, Morrison JL, McMillen IC. The transition from fetal growth restriction to accelerated postnatal growth: a potential role for insulin signalling in skeletal muscle. J Physiol. 2009;587(Pt 17):4199-211. doi: 10.1113/jphysiol.2009.173161. Epub 2009 Jul 21. | Animal |
| 141 | Alexander KE, Ventura EE, Spruijt-Metz D, Weigensberg MJ, Goran MI, Davis JN. Association of breakfast skipping with visceral fat and insulin indices in overweight Latino youth. Obesity (Silver Spring). 2009;17(8):1528-33. doi: 10.1038/oby.2009.127. Epub 2009 May 7. | Dupl 47PM 38Sc |
| 142 | Abete I, Parra D, Crujeiras AB, Goyenechea E, Martinez JA. Specific insulin sensitivity and leptin responses to a nutritional treatment of obesity via a combination of energy restriction and fatty fish intake. J Hum Nutr Diet. 2008;21(6):591-600. doi: 10.1111/j.1365-277X.2008.00902.x. Epub 2008 Aug 27. | Unrelated |
| 143 | Venu L, Padmavathi IJ, Kishore YD, Bhanu NV, Rao KR, Sainath PB, Ganeshan M, Raghunath M. Long-term effects of maternal magnesium restriction on adiposity and insulin resistance in rat pups. Obesity (Silver Spring). 2008;16(6):1270-6. doi: 10.1038/oby.2008.72. Epub 2008 Mar 27. | Animal |
| 144 | Takii M, Uchigata Y, Tokunaga S, Amemiya N, Kinukawa N, Nozaki T, Iwamoto Y, Kubo C. The duration of severe insulin omission is the factor most closely associated with the microvascular complications of Type 1 diabetic females with clinical eating disorders. Int J Eat Disord. 2008;41(3):259-64. doi: 10.1002/eat.20498. | Dupl 50PM |
| 145 | Ruivo GF, Leandro SM, do Nascimento CA, Catanozi S, Rocha JC, Furukawa LN, Dolnikoff MS, Quintão EC, Heimann JC. Insulin resistance due to chronic salt restriction is corrected by alpha and beta blockade and by L-arginine. Physiol Behav. 2006;88(4-5):364-70. doi: 10.1016/j.physbeh.2006.04.006. Epub 2006 Jun 5. | Animal |
| 146 | Takii M, Komaki G, Uchigata Y, Maeda M, Omori Y, Kubo C. Differences between bulimia nervosa and binge-eating disorder in females with type 1 diabetes: the important role of insulin omission. J Psychosom Res. 1999;47(3):221-31. doi: 10.1016/s0022-3999(99)00031-8. | Dupl 56PM |
| 147 | Khan Y, Montgomery AMJ. Eating attitudes in young females with diabetes: insulin omission identifies a vulnerable subgroup. Br J Med Psychol. 1996;69(4):343-53. doi: 10.1111/j.2044-8341.1996.tb01877.x. | Dupl PM57 Sc47 |
| 148 | Hell NS, Costa de Olivera LB, Dolnikoff MS, Scivoletto R, Timo-Iaria C. Changes of carbohydrate metabolism caused by food restriction, as detected by insulin administration. Physiol Behav. 1980;24(3):473-7. doi: 10.1016/0031-9384(80)90239-5. | Unrelated |
| 149 | Christofferson JL, Okonak K, Kazak AE, Pierce J, Kelly C, Schifano E, Sciolla J, Deatrick JA, Alderfer MA. Family consequences of potentially traumatic pediatric medical events: Implications for trauma-informed care. J Fam Psychol. 2020 Mar;34(2):237-246. doi: 10.1037/fam0000597. Epub 2019 Oct 24. | Unrelated |
| 150 | Mantantzis K, Drewelies J, Duezel S, Steinhagen-Thiessen E, Demuth I, Wagner GG, Lindenberger U, Gerstorf D. Dehydration predicts longitudinal decline in cognitive functioning and well-being among older adults. Psychol Aging. 2020 Jun;35(4):517-528. doi: 10.1037/pag0000471. Epub 2020 Apr 30. | Unrelated |
| TI diabulimia OR TI "insulin restriction" OR TI "skipping insulin" OR TI "insulin omission" 1.4.2026 CINAHL → 28 Documents | | |
| 151 | Oikonomou A, Christoforidis A, Kotanidou EP, Giannopoulou I, Paschalidou E, Tsinopoulou VR, Sotiriou G, Tsiroukidou K, Galli-Tsinopoulou A. Detecting Disordered Eating Behaviors in Greek Youth with Type 1 Diabetes Mellitus by Using the Diabetes Eating Problem Survey-Revised (DEPS-R): Associations with Insulin Restriction, Glycemic Control, and Anthropometric Parameters. Children (Basel). 2025;12(6):795. doi: 10.3390/children12060795. | Dupl 2PM |
| 152 | Beam AB, Wiebe DJ. Subtypes of Insulin Restriction in Diabetes Management: A Systematic Review. Curr Diab Rep. 2025;25(1):20. doi: 10.1007/s11892-025-01577-3. | Dupl 5PM 1Sc |
| 153 | Ng SJY, Goh ML. Reducing insulin omission errors among patients with diabetes mellitus in general surgical wards: a best practice implementation project. JBI Evid Implement. 2024;22(3):291-302. doi: 10.1097/XEB.0000000000000437. | Dupl 8PM |
| 154 | Levek N, Faruge-Hadiga R, Pinhas-Hamiel O. Insulin Omission for Weight Loss in a Female Adolescent Treated With Advanced Hybrid Closed-Loop System: A Word of Caution. Diabetes Care. 2023;46(8):e143-e145. doi: 10.2337/dc23-0204. | Dupl 15PM |
| 155 | Goddard G, Oxlad M. Insulin restriction or omission in Type 1 Diabetes Mellitus: a meta-synthesis of individuals' experiences of diabulimia. Health Psychol Rev. 2023;17(2):227-246. doi: 10.1080/17437199.2021.2025133. Epub 2022 Jan 31. | Dupl 19PM 8Sc 8Ps |
| 156 | Welsh, Erin T. Disordered eating, insulin restriction reduce beneficial gut microbes. Endocrine Today, 02/01/2023. | Opinion |
| 157 | Beam AB, Wiebe DJ, Berg CA. Insulin Restriction, Emotion Dysregulation, and Depressive Symptoms in Late Adolescents with Diabetes. J Pediatr Psychol. 2021;46(9):1110-1118. doi: 10.1093/jpepsy/jsab042. | Dupl 20PM 11Sc 10Ps |
| 158 | Hall R, Keeble L, Sünram-Lea SI, To M. A review of risk factors associated with insulin omission for weight loss in type 1 diabetes. Clin Child Psychol Psychiatry. 2021;26(3):606-616. doi: 10.1177/13591045211026142. Epub 2021 Jun 13. | Dupl 21PM 11Ps |
| **159** | **Papadakis JL, Anderson LM, Vesco AT, Evans MA, Weissberg-Benchell J. 207-OR: Intentional Insulin Omission for Weight Loss and Psychosocial Outcomes among Youth with Type 1 Diabetes: Findings from Routine Screening. Diabetes 2019;68(Suppl.1):207–OR. doi: 10.2337/db19-207-OR** | **Included** |
| 160 | De Paoli T, Rogers PJ. Disordered eating and insulin restriction in type 1 diabetes: A systematic review and testable model. Eat Disord. 2018;26(4):343-360. doi: 10.1080/10640266.2017.1405651. Epub 2017 Nov 28. | Dupl 29 24Sc 14Ps |
| 161 | Brookes G. Insulin restriction, medicalisation and the Internet. Commun Med. 2019;15(1):14-27. doi: 10.1558/cam.33067. | Dupl 26 |

| | | |
|---|---|---|
| 162 | Wisting L, Reas DL, Bang L, Skrivarhaug T, Dahl-Jørgensen K, Rø Ø. Eating patterns in adolescents with type 1 diabetes: Associations with metabolic control, insulin omission, and eating disorder pathology. Appetite. 2017;114:226-231. doi: 10.1016/j.appet.2017.03.035. Epub 2017 Mar 27. | Dupl 33PM 26Sc 16Ps |
| 163 | Bächle C, Stahl-Pehe A, Rosenbauer J. Disordered eating and insulin restriction in youths receiving intensified insulin treatment: Results from a nationwide population-based study. Int J Eat Disord. 2016;49(2):191-6. doi: 10.1002/eat.22463. Epub 2015 Sep 23. | Dupl 36 18Ps |
| 164 | Merwin RM, Dmitrieva NO, Honeycutt LK, Moskovich AA, Lane JD, Zucker NL, Surwit RS, Feinglos M, Kuo J. Momentary Predictors of Insulin Restriction Among Adults With Type 1 Diabetes and Eating Disorder Symptomatology. Diabetes Care. 2015;38(11):2025-32. doi: 10.2337/dc15-0753. Epub 2015 Sep 17. | Dupl 37 |
| 165 | Gottesman, Kimberly; Ziegler, Jane;Parker, Anna. Insulin Omission for Weight Control in Adolescents With Type 1 Diabetes Mellitus. Top Clin Nutr. 2015;30(4):314-323. doi: 10.1097/TIN.0000000000000050. | Review |
| 166 | Allan, Jacqueline; Nash, Jen. Diabetes and eating disorders: Insulin omission and the DSM-5. J Diab Nurs. 2014;18(9):386-387. | Opinion |
| 167 | Pinhas-Hamiel O, Hamiel U, Greenfield Y, Boyko V, Graph-Barel C, Rachmiel M, Lerner-Geva L, Reichman B. Detecting intentional insulin omission for weight loss in girls with type 1 diabetes mellitus. Int J Eat Disord. 2013;46(8):819-25. doi: 10.1002/eat.22138. Epub 2013 May 15. | Dupl 39PM 22Ps |
| 168 | Editorial. Insulin restriction in women with T1D. Diabetes Digest, 2011;10(3):136. | Opinion |
| 169 | Murdoff L. Insulin omission for weight loss. The dangers of diabulimia. Adv NPs PAs. 2011;2(5):35-7, 50. | Dupl 41 |
| 170 | Goebel-Fabbri AE, Anderson BJ, Fikkan J, Franko DL, Pearson K, Weinger K. Improvement and emergence of insulin restriction in women with type 1 diabetes. Diabetes Care. 2011;34(3):545-50. doi: 10.2337/dc10-1547. Epub 2011 Jan 25. | Dupl 43 |
| 171 | Haagen BF. Insulin omission. A troubling trend among adolescent girls. J Psychosoc Nurs Ment Health Serv. 2011;49(2):6-7. doi: 10.3928/02793695-20110116-01. | Dupl 42PM 25Ps |
| 172 | Roberts S. Diabetics skipping insulin to lose kilos. Pharmacy News, 20/11/2008, p. 3. | Opinion |
| 173 | Goebel-Fabbri AE. Diabulimia: insulin restriction, eating disorders and type 1 diabetes. Paradigm (Targeted Publications Group, Inc), 2008 Fall, 13(4): 8-9. | Opinion |
| 174 | Takii M, Uchigata Y, Tokunaga S, Amemiya N, Kinukawa N, Nozaki T, Iwamoto Y, Kubo C. The duration of severe insulin omission is the factor most closely associated with the microvascular complications of Type 1 diabetic females with clinical eating disorders. Int J Eat Disord. 2008;41(3):259-64. doi: 10.1002/eat.20498. | Dupl 50PM 31Ps |
| 175 | Goebel-Fabbri AE, Fikkan J, Franko DL, Pearson K, Anderson BJ, Weinger K. Insulin restriction and associated morbidity and mortality in women with type 1 diabetes. Diabetes Care. 2008;31(3):415-9. doi: 10.2337/dc07-2026. Epub 2007 Dec 10. | Dupl 51PM |
| 176 | Editorial. Increased mortality with insulin restriction. Diabetes Professional, 2008 Spring | Opinion |
| 177 | Affenito SG, Adams CH. Are eating disorders more prevalent in females with type 1 diabetes mellitus when the impact of insulin omission is considered? Nutr Rev. 2001;59(6):179-82. doi: 10.1111/j.1753-4887.2001.tb07010.x. | Dupl 55PM |
| 178 | Polonsky WH, Anderson BJ, Lohrer PA, Aponte JE, Jacobson AM, Cole CF. Insulin omission in women with IDDM. Diabetes Care. 1994;17(10):1178-85. doi: 10.2337/diacare.17.10.1178. | Dupl 58PM |
| ClinicalTrials.gov 1.4.2026 Condition/Disease: diabulimia OR Other terms: intentional insulin omission → 0 documents | | |
| Other sources → 11 documents | | |
| 179 | Goebel-Fabbri AE. Diabetes and eating disorders. J Diabetes Sci Technol. 2008;2(3):530-2. doi: 10.1177/193229680800200326. | Opinion |
| 180 | Lorettu L, Pes GM, Dore MP, Milia P, Nivoli A. Eating disorders and diabetes: behavioural patterns and psychopathology. Two case reports. Riv Psichiatr. 2020;55(4):240-244. doi: 10.1708/3417.34001. | Case |
| 181 | Gamble J-M, Simpson SH, Eurich DT, Majumdar SR, Johnson JA. Insulin use and increased risk of mortality in type 2 diabetes: a cohort study. Diabetes Obes Metab. 2010;12(1):47-53. doi: 10.1111/j.1463-1326.2009.01125.x. Epub 2009 Sep 24. | Unfocused |
| 182 | Yahia S, Salem NA, Tobar S, Abdelmoneim Z, Mahmoud AM, Laimon W. Shedding light on eating disorders in adolescents with type 1 diabetes: insights and implications. Eur J Pediatr. 2025;184(4):272. doi: 10.1007/s00431-025-06081-0. | Unfocused |
| 183 | Amy Shelford A, Tomlin A, Wood L. Interventions for insulin omission for weight loss in people with type 1 diabetes without a clinical eating disorder: a systematic review. Preview DOI:10.31234/osf.io/nkdc8. | Review |
| **184** | **Igudesman D, Crandell J, Corbin KD, Zaharieva DP, Addala A, Thomas JM, Bulik CM, Pence BW, Pratley RE, Kosorok MR, Maahs DM, Carroll IM, Mayer-Davis EJ. Associations of disordered eating with the intestinal microbiota and short-chain fatty acids among young adults with type 1 diabetes. Nutr Metab Cardiovasc Dis. 2023;33(2):388-398. doi: 10.1016/j.numecd.2022.11.017. Epub 2022 Nov 17.** | **Included** |
| **185** | **Tarçın G, Akman H, Güneş Kaya D, Serdengeçti N, İncetahtacı S, Turan H, Doğangün B, Ercan O. Diabetes-specific eating disorder and possible associated psychopathologies in adolescents with type 1 diabetes mellitus. Eat Weight Disord. 2023;28(1):36. doi: 10.1007/s40519-023-01559-y. Erratum in: Eat Weight Disord. 2023;28(1):48. doi: 10.1007/s40519-023-01576-x.** | **Included** |
| 186 | Gade, Alira Bianca Mae 2022 The Truth about Diabulimia – A scoping review on the experiences of type 1 diabetic patients with Diabulimia. Degree Thesis in Health Care and Social Welfare, Education: Bachelor of Health Care, Nursing, Novia University of Applied Sciences, Vaasa, Turku, Raasepori, and Pietarsaari. 2022. | Review |
| **187** | **Şahin-Bodur G, Keser A, Şıklar Z, Berberoğlu M. Determining the risk of diabulimia and its relationship with diet quality and nutritional status of adolescents with type 1 diabetes [Déterminer le risque de diaboulimie et sa relation avec la qualité de l'alimentation et l'état nutritionnel des adolescents atteints de diabète de type 1]. Nutrition Clinique et Métabolisme. 2021;35(4):281-7. doi: 10.1016/j.nupar.2021.05.002.** | **Included** |
| 188 | Evdokimova NV, Kaplina DK. Евдокимова Н.В., Каплина Д.К. Диабулимия-расстройствопищевогоповеденияпервоготипа (обзор литературы). [Diabulimia – Eating Disorder Type I. (literature review)]. Научно-медицинский вестник Центрального Черноземья. [Scientific and Medical Bulletin of the Central Black Earth Region]. 2025;(1):55-63. https://doi.org/10.18499/1990-472X-2025-26-1-55-63. | Review |
| 189 | Callum AM., Lewis LM. Diabulimia among adolescents and young adults with Type 1 diabetes. Clin Nurs Stud. 2014;2(4):12-16. https://doi.org/10.5430/cns.v2n4p12. | Review |

Included 29

Excluded 160

| | |
|---|---|
| Review | 30 |
| Opinion | 16 |
| Case | 13 |
| Lumping | 0 |
| Overlap | 0 |
| Animal | 11 |
| No patients | 3 |
| No diabetes | 1 |
| No diabulimia | 1 |
| Unfocused | 10 |
| Unrelated | 26 |
| Duplicates | 49 |

Supplementary Table S2. PRISMA Checklist.

| Section and Topic | Item # | Checklist item | Location where item is reported |
|---|---|---|---|
| **TITLE** | | | 1 |
| Title | 1 | Identify the report as a systematic review. | 1 |
| **ABSTRACT** | | | 1 |
| Abstract | 2 | See the PRISMA 2020 for Abstracts checklist. | 1 |
| **INTRODUCTION** | | | 2 |
| Rationale | 3 | Describe the rationale for the review in the context of existing knowledge. | 2 |
| Objectives | 4 | Provide an explicit statement of the objective(s) or question(s) the review addresses. | 2 |
| **METHODS** | | | 3 |
| Eligibility criteria | 5 | Specify the inclusion and exclusion criteria for the review and how studies were grouped for the syntheses. | 3 |
| Information | 6 | Specify all databases, registers, websites, organisations, reference lists and other sources searched or consulted to | 3 |

| Section and Topic | Item # | Checklist item | Location where item is reported |
|---|---|---|---|
| sources | | identify studies. Specify the date when each source was last searched or consulted. | |
| Search strategy | 7 | Present the full search strategies for all databases, registers and websites, including any filters and limits used. | 3 |
| Selection process | 8 | Specify the methods used to decide whether a study met the inclusion criteria of the review, including how many reviewers screened each record and each report retrieved, whether they worked independently, and if applicable, details of automation tools used in the process. | 3 |
| Data collection process | 9 | Specify the methods used to collect data from reports, including how many reviewers collected data from each report, whether they worked independently, any processes for obtaining or confirming data from study investigators, and if applicable, details of automation tools used in the process. | 3 |
| Data items | 10a | List and define all outcomes for which data were sought. Specify whether all results that were compatible with each outcome domain in each study were sought (e.g. for all measures, time points, analyses), and if not, the methods used to decide which results to collect. | 3 |
| | 10b | List and define all other variables for which data were sought (e.g. participant and intervention characteristics, funding sources). Describe any assumptions made about any missing or unclear information. | 3 |
| Study risk of bias assessment | 11 | Specify the methods used to assess risk of bias in the included studies, including details of the tool(s) used, how many reviewers assessed each study and whether they worked independently, and if applicable, details of automation tools used in the process. | 3 |
| Effect measures | 12 | Specify for each outcome the effect measure(s) (e.g. risk ratio, mean difference) used in the synthesis or presentation of results. | 3 |
| Synthesis methods | 13a | Describe the processes used to decide which studies were eligible for each synthesis (e.g. tabulating the study intervention characteristics and comparing against the planned groups for each synthesis (item #5)). | 3 |
| | 13b | Describe any methods required to prepare the data for presentation or synthesis, such as handling of missing summary statistics, or data conversions. | 3 |
| | 13c | Describe any methods used to tabulate or visually display results of individual studies and syntheses. | 3 |
| | 13d | Describe any methods used to synthesize results and provide a rationale for the choice(s). If meta-analysis was performed, describe the model(s), method(s) to identify the presence and extent of statistical heterogeneity, and software package(s) used. | 3 |
| | 13e | Describe any methods used to explore possible causes of heterogeneity among study results (e.g. subgroup analysis, meta-regression). | 3 |
| | 13f | Describe any sensitivity analyses conducted to assess robustness of the synthesized results. | 3 |
| Reporting bias assessment | 14 | Describe any methods used to assess risk of bias due to missing results in a synthesis (arising from reporting biases). | 3 |
| Certainty assessment | 15 | Describe any methods used to assess certainty (or confidence) in the body of evidence for an outcome. | 3 |
| **RESULTS** | | | 4-18 |
| Study selection | 16a | Describe the results of the search and selection process, from the number of records identified in the search to the number of studies included in the review, ideally using a flow diagram. | 4 |
| | 16b | Cite studies that might appear to meet the inclusion criteria, but which were excluded, and explain why they were excluded. | 4 (Suppl.) |
| Study characteristics | 17 | Cite each included study and present its characteristics. | 4-15 |
| Risk of bias in studies | 18 | Present assessments of risk of bias for each included study. | 16-18 |
| Results of individual studies | 19 | For all outcomes, present, for each study: (a) summary statistics for each group (where appropriate) and (b) an effect estimate and its precision (e.g. confidence/credible interval), ideally using structured tables or plots. | N/A |
| Results of syntheses | 20a | For each synthesis, briefly summarise the characteristics and risk of bias among contributing studies. | 16-18 |
| | 20b | Present results of all statistical syntheses conducted. If meta-analysis was done, present for each the summary estimate and its precision (e.g. confidence/credible interval) and measures of statistical heterogeneity. If comparing groups, describe the direction of the effect. | N/A |
| | 20c | Present results of all investigations of possible causes of heterogeneity among study results. | 4, 25 |
| | 20d | Present results of all sensitivity analyses conducted to assess the robustness of the synthesized results. | N/A |
| Reporting biases | 21 | Present assessments of risk of bias due to missing results (arising from reporting biases) for each synthesis assessed. | 16-18 |
| Certainty of evidence | 22 | Present assessments of certainty (or confidence) in the body of evidence for each outcome assessed. | N/A |
| **DISCUSSION** | | | 18-26 |
| Discussion | 23a | Provide a general interpretation of the results in the context of other evidence. | 18 |
| | 23b | Discuss any limitations of the evidence included in the review. | 25-26 |
| | 23c | Discuss any limitations of the review processes used. | 25-26 |
| | 23d | Discuss implications of the results for practice, policy, and future research. | 24-26 |
| **OTHER INFORMATION** | | | 26 |
| Registration and protocol | 24a | Provide registration information for the review, including register name and registration number, or state that the review was not registered. | 3 |
| | 24b | Indicate where the review protocol can be accessed, or state that a protocol was not prepared. | 3 |
| | 24c | Describe and explain any amendments to information provided at registration or in the protocol. | N/A |
| Support | 25 | Describe sources of financial or non-financial support for the review, and the role of the funders or sponsors in the review. | 26 |
| Competing interests | 26 | Declare any competing interests of review authors. | 26 |
| Availability of data, code and other materials | 27 | Report which of the following are publicly available and where they can be found: template data collection forms; data extracted from included studies; data used for all analyses; analytic code; any other materials used in the review. | 26, N/A |

*From:* Page MJ, McKenzie JE, Bossuyt PM, Boutron I, Hoffmann TC, Mulrow CD, et al. The PRISMA 2020 statement: an updated guideline for reporting systematic reviews. BMJ 2021;372:n71. doi: 10.1136/bmj.n71. This work is licensed under CC BY 4.0. To view a copy of this license, visit https://creativecommons.org/licenses/by/4.0/

PRISMA

## PRISMA 2020 for Abstracts Checklist

| Section and Topic | Item # | Checklist item | Reported (Yes/No) |
|---|---|---|---|
| **TITLE** | | | |
| Title | 1 | Identify the report as a systematic review. | Y |

| Section and Topic | Item # | Checklist item | Reported (Yes/No) |
|---|---|---|---|
| **BACKGROUND** | | | |
| Objectives | 2 | Provide an explicit statement of the main objective(s) or question(s) the review addresses. | Y |
| **METHODS** | | | |
| Eligibility criteria | 3 | Specify the inclusion and exclusion criteria for the review. | N |
| Information sources | 4 | Specify the information sources (e.g. databases, registers) used to identify studies and the date when each was last searched. | Y |
| Risk of bias | 5 | Specify the methods used to assess risk of bias in the included studies. | N (yes in MS) |
| Synthesis of results | 6 | Specify the methods used to present and synthesise results. | Y |
| **RESULTS** | | | |
| Included studies | 7 | Give the total number of included studies and participants and summarise relevant characteristics of studies. | Y |
| Synthesis of results | 8 | Present results for main outcomes, preferably indicating the number of included studies and participants for each. If meta-analysis was done, report the summary estimate and confidence/credible interval. If comparing groups, indicate the direction of the effect (i.e. which group is favoured). | N (yes in manuscript) |
| **DISCUSSION** | | | |
| Limitations of evidence | 9 | Provide a brief summary of the limitations of the evidence included in the review (e.g. study risk of bias, inconsistency and imprecision). | N |
| Interpretation | 10 | Provide a general interpretation of the results and important implications. | Y |
| **OTHER** | | | |
| Funding | 11 | Specify the primary source of funding for the review. | N/A |
| Registration | 12 | Provide the register name and registration number. | N (yes in MS) |

From: Page MJ, McKenzie JE, Bossuyt PM, Boutron I, Hoffmann TC, Mulrow CD, et al. The PRISMA 2020 statement: an updated guideline for reporting systematic reviews. BMJ 2021;372:n71. doi: 10.1136/bmj.n71. This work is licensed under CC BY 4.0. To view a copy of this license, visit https://creativecommons.org/licenses/by/4.0/

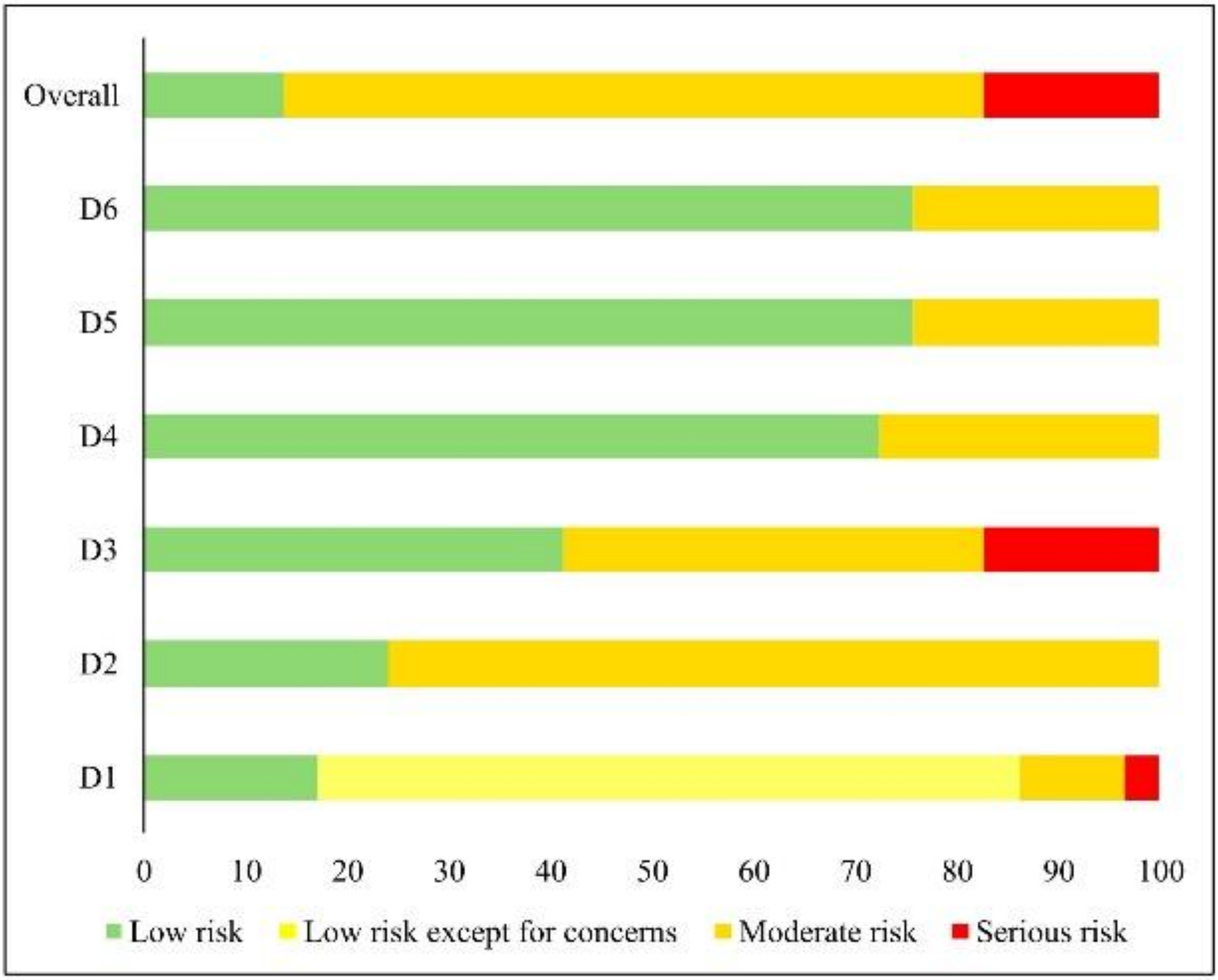


**Figure S1.** Risk of Bias assessment using the ROBINS-I V2 tool for all included records.
